# LSD1 promotes prostate cancer reprogramming by repressing TP53 signaling independently of its demethylase function

**DOI:** 10.1172/jci.insight.167440

**Published:** 2023-08-08

**Authors:** Anbarasu Kumaraswamy, Zhi Duan, Diana Flores, Chao Zhang, Archana Sehrawat, Ya-Mei Hu, Olivia A. Swaim, Eva Rodansky, William K. Storck, Joshua A. Kuleape, Karan Bedi, Rahul Mannan, Xiao-Ming Wang, Aaron Udager, Visweswaran Ravikumar, Armand Bankhead, Ilsa Coleman, John K. Lee, Colm Morrissey, Peter S. Nelson, Arul M. Chinnaiyan, Arvind Rao, Zheng Xia, Joel A. Yates, Joshi J. Alumkal

**Affiliations:** 1 Department of Internal Medicine and; 2 Rogel Cancer Center, University of Michigan, Ann Arbor, Michigan, USA.; 3 Knight Cancer Institute and; 4 Biomedical Engineering Department, Oregon Health & Science University (OHSU), Portland, Oregon, USA.; 5 College of Literature, Science, and the Arts, and; 6 Department of Biostatistics, School of Public Health, University of Michigan, Ann Arbor, Michigan, USA.; 7 Department of Pathology, University of Michigan Medical School, Ann Arbor, Michigan, USA.; 8 Michigan Center for Translational Pathology, Ann Arbor, Michigan, USA.; 9 Department of Computational Medicine & Bioinformatics, University of Michigan, Ann Arbor, Michigan, USA.; 10 Divisions of Human Biology and Clinical Research, Fred Hutchinson Cancer Research Center, Seattle, Washington, USA.; 11 Department of Urology, University of Washington, Seattle, Washington, USA.; 12 Department of Urology, University of Michigan Medical School, Ann Arbor, Michigan, USA.; 13 Howard Hughes Medical Institute, Ann Arbor, Michigan, USA.; 14 Department of Radiation Oncology and; 15 Department of Biomedical Engineering, University of Michigan, Ann Arbor, Michigan, USA.

**Keywords:** Oncology, Epigenetics, Prostate cancer, p53

## Abstract

Lysine-specific demethylase 1 (LSD1) is a histone demethylase that promotes stemness and cell survival in cancers such as prostate cancer. Most prostate malignancies are adenocarcinomas with luminal differentiation. However, some tumors undergo cellular reprogramming to a more lethal subset termed neuroendocrine prostate cancer (NEPC) with neuronal differentiation. The frequency of NEPC is increasing since the widespread use of potent androgen receptor signaling inhibitors. Currently, there are no effective treatments for NEPC. We previously determined that LSD1 promotes survival of prostate adenocarcinoma tumors. However, the role of LSD1 in NEPC is unknown. Here, we determined that LSD1 is highly upregulated in NEPC versus adenocarcinoma patient tumors. LSD1 suppression with RNAi or allosteric LSD1 inhibitors — but not catalytic inhibitors — reduced NEPC cell survival. RNA-Seq analysis revealed that LSD1 represses pathways linked to luminal differentiation, and *TP53* was the top reactivated pathway. We confirmed that LSD1 suppressed the *TP53* pathway by reducing TP53 occupancy at target genes while LSD1’s catalytic function was dispensable for this effect. Mechanistically, LSD1 inhibition disrupted LSD1-HDAC interactions, increasing histone acetylation at TP53 targets. Finally, LSD1 inhibition suppressed NEPC tumor growth in vivo. These findings suggest that blocking LSD1’s noncatalytic function may be a promising treatment strategy for NEPC.

## Graphical abstract

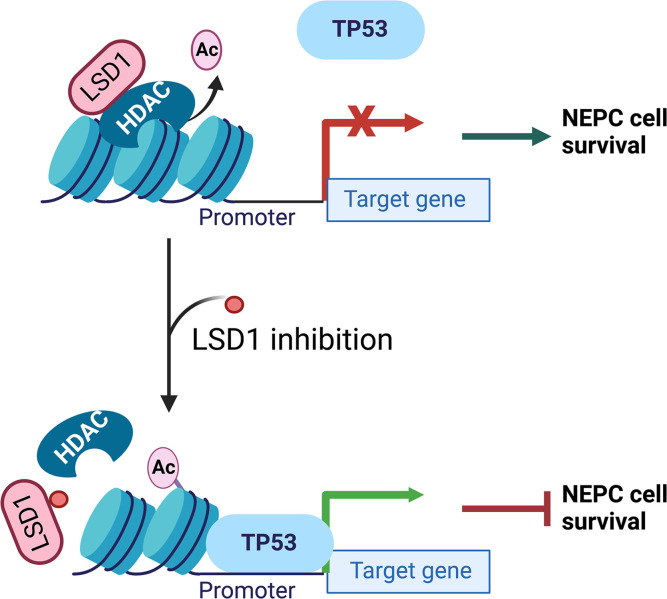

## Introduction

Prostate cancer is the second leading cause of cancer-related death in men in the United States, with 34,700 men predicted to die of this disease in 2023 (1). At diagnosis, nearly all prostate cancers are adenocarcinomas driven by androgen receptor (AR) signaling. In addition to promoting the proliferation of prostate cancer cells, the AR also promotes a luminal differentiation program (2). AR interference — including treatment with potent, AR-signaling inhibitors (ARSIs) — is the standard therapy once prostate cancers metastasize. Since the widespread use of these ARSIs, the frequency of prostate tumors that have reduced reliance on the AR and activation of alternative differentiation programs has increased (3, 4). The most aggressive example is neuroendocrine prostate cancer (NEPC) (3, 5).

NEPC is associated with poor patient outcomes, and there are currently no effective therapies (3). Thus, there is an urgent need to identify vulnerabilities in NEPC. Genetic inactivation of the tumor suppressors *PTEN*, *RB1*, and *TP53* are commonly found in NEPC (5). Previous studies have determined that loss of tumor suppressors *RB1* and *TP53* promotes nonluminal differentiation programs, including NEPC (6, 7). In addition to genetic loss of *TP53* function by deletion or mutation, TP53 protein can also be suppressed through nongenetic mechanisms (7, 8). Finally, activation of several additional factors — such as N-Myc, E2F1, EZH2, or SOX2 — promotes cellular reprogramming and NEPC differentiation (9–12).

Our prior work identified lysine-specific demethylase 1 (LSD1; KDM1A) as a key driver promoting AR-independent survival of prostate adenocarcinoma (PRAD) cells (13). LSD1’s scaffold function — rather than its catalytic function — appeared to be most crucial for LSD1’s growth-promoting effects (13). In addition to prostate cancer, LSD1 is a key driver in multiple cancers, including Merkel cell carcinoma, acute myeloid leukemia, squamous cell carcinoma, small cell lung cancer, and neuroblastoma (14–17). Targeting LSD1 activates tumor suppressor pathways and immunomodulatory pathways in multiple cancers (16–19). In small cell lung cancer, which has similar genomic and phenotypic features as NEPC, LSD1 inhibition blocks tumor progression (17, 20). These results prompted us to investigate LSD1’s role in reprogrammed cells, including NEPC.

In this report, we determined that LSD1 is highly expressed in NEPC patient samples and that LSD1 promotes the proliferation of NEPC tumors. LSD1 inhibition reactivates the *TP53* pathway in multiple NEPC cell models, and induction of TP53 signaling is critical for the antitumor effects of LSD1 inhibition. We determined that LSD1’s catalytic function appears to be dispensable for repressing TP53 signaling and promoting NEPC cell survival, and LSD1 inhibition with RNA interference (RNAi) or an allosteric LSD1 inhibitor reduces NEPC tumor growth in vivo. Together, our results demonstrate that LSD1 is worthy of further study as a therapeutic target in reprogrammed prostate cancer cells, including NEPC.

## Results

### LSD1 is upregulated in NEPC and is important for NEPC cell survival.

We determined previously that LSD1 was more highly expressed in metastatic castration-resistant prostate cancer (CRPC) progressing on androgen-lowering treatments — the vast majority of which were adenocarcinomas — versus localized prostate cancer (13). However, there was little information with respect to LSD1 expression in NEPC versus adenocarcinoma tumors. Therefore, we examined LSD1 expression in 2 CRPC patient cohorts that included both NEPC and adenocarcinoma tumors (3, 5). *LSD1* mRNA was upregulated in NEPC versus adenocarcinoma in both the Beltran et al. (5) and Aggarwal et al. (3) data sets (Supplemental Figure 1, A and B; supplemental material available online with this article; https://doi.org/10.1172/jci.insight.167440DS1). We next analyzed *LSD1* expression in *Pten/Rb1/Trp53*-KO genetically engineered mouse models developed by Ku et al. that recapitulate human NEPC tumors (6). *LSD1* was significantly upregulated in the NEPC tumors with *Pten*/*Rb1* double knockout (DKO), castration-resistant DKO (DKO-Cr), or *Pten*/*Rb1*/*Trp53* triple knockout (TKO) (Supplemental Figure 1C). We also examined *LSD1* expression in the LTL331→LTL331R patient-derived xenograft (PDX) model of castration-induced NEPC transdifferentiation developed by Lin et al. (21). *LSD1* mRNA was increased in the progression LTL331R NEPC tumors versus baseline LTL331 adenocarcinoma tumors (Supplemental Figure 1D). These data demonstrate that *LSD1* is upregulated in tumors undergoing NEPC reprogramming.

**Figure 1:**
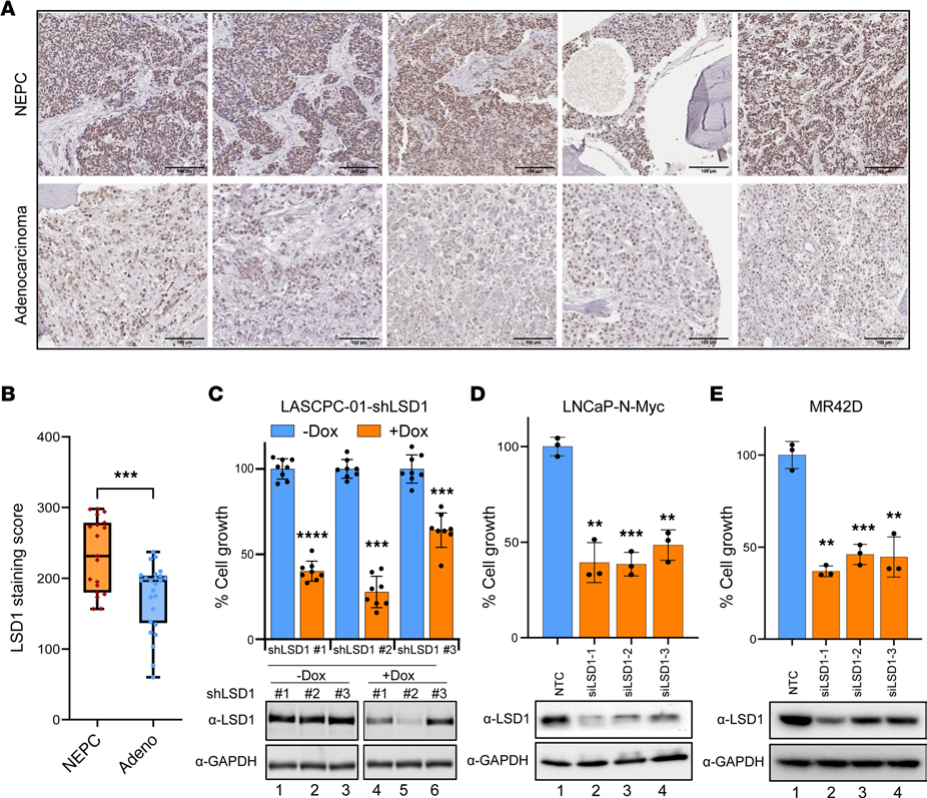
LSD1 is upregulated in NEPC and promotes NEPC cell survival. (**A**) Prostate cancer patient tumor samples were stained with an anti-LSD1 antibody. Representative images from LSD1-stained tumor samples are shown. Scale bar: 100 μm. (**B**) The intensity of LSD1 staining was evaluated as product scores to quantify LSD1 protein expression in NEPC (*n* = 19) and adenocarcinoma (*n* = 25) cohorts. Data are presented in box plots with the median indicated, boxes representing the 25th-75th percentiles, and whiskers representing minimum to maximum values. (**C**) LASCPC-01 cells were transduced with lentiviral doxycycline-inducible (dox-inducible) shLSD1. Cell viability was measured 5 days after induction with vehicle or dox (500 ng/mL) (top), *n* = 8. Data are reported as the mean ± SD. Knockdown of LSD1 was confirmed by Western blot analysis of cell lysates normalized to GAPDH (bottom). (**D** and **E**) LNCaP–N-Myc (**D**) or MR42D (**E**) NEPC cell lines were transfected with nontargeting control (NTC) or 3 different siLSD1 siRNAs, and cell viability was measured 96 hours after transfection (top), *n* = 3. Data are reported as the mean ± SD. Knockdown of LSD1 was confirmed by Western blot analysis of cell lysates normalized to GAPDH (bottom). For **C**–**E** statistical analysis, unpaired 2-tailed Welch’s *t* tests were performed. **P < 0.01, ***P < 0.001, ****P < 0.0001.

We next sought to understand whether *LSD1* expression correlates with loss of AR signaling and gain of a neuroendocrine program. Therefore, we analyzed 3 different patient cohorts (5, 22, 23) and examined the correlation between LSD1 expression and AR function using a previously described AR activity signature (ARG10) (24) or NEPC differentiation using a signature of genes highly upregulated in NEPC described by Beltran et al. (5). LSD1 expression was negatively correlated with the AR activity signature and positively correlated with the NEPC signature, suggesting that LSD1 upregulation is linked to AR activity–low tumors, including those that have an NEPC program (Supplemental Figure 1E). Next, we measured LSD1 protein expression with IHC and determined that LSD1 protein expression was significantly elevated in NEPC versus adenocarcinoma tumors (Figure 1, A and B, and Supplemental Table 1). Because of these results, we hypothesized that LSD1 may play an important role in NEPC.

To determine the importance of LSD1 for NEPC cell survival, we suppressed LSD1 using either shRNA or siRNA in cell models of NEPC, including: LASCPC-01, normal basal prostate epithelial cells transduced with constitutively activated Akt and N-Myc (10); LNCaP–N-Myc, LNCaP cells with constitutive overexpression of the N-Myc oncogene (9); and MR42D, an enzalutamide-resistant, LNCaP-derived treatment-emergent NEPC (t-NEPC) model (25). LSD1 knockdown significantly reduced cell viability in each model, demonstrating LSD1’s importance for NEPC cell growth (Figure 1, C–E). Several of the NEPC models we used express N-Myc. We next sought to determine whether N-Myc regulates LSD1 expression. We tested LSD1 expression upon overexpression of N-Myc in adenocarcinoma models or upon N-Myc knockdown in an NEPC model. These results demonstrate that N-Myc does not modulate LSD1 expression (Supplemental Figure 1, F and G).

### Catalytic function of LSD1 is dispensable to promote NEPC cell survival.

**Figure 2:**
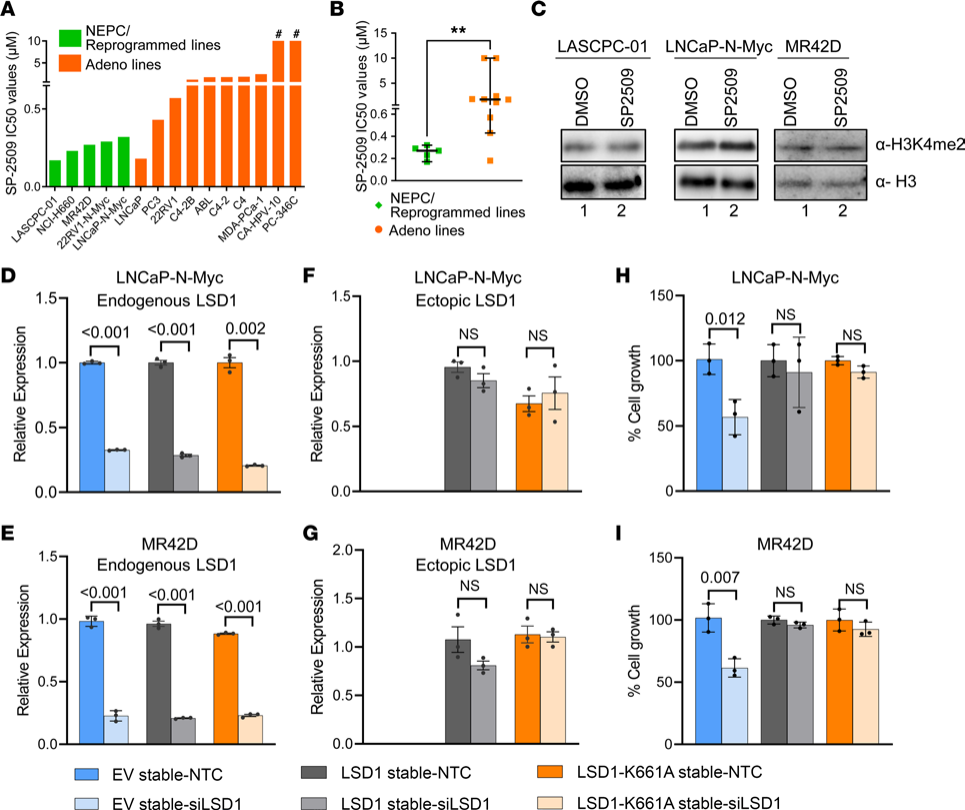
LSD1’s catalytic function is dispensable for promoting NEPC cell survival. (**A**) SP2509 was tested by dose response in a panel of prostate cancer cell lines for 72 hours. Cell viability was measured by CTG. IC_50_ values are shown. The # symbol indicates that CA-HPV-10 and PC-346C did not reach IC_50_ using doses up to 10 μM of SP2509. (**B**) IC_50_ values of NEPC and adenocarcinoma cell line models in NEPC cell lines (green) and adenocarcinoma cell lines (orange). Data are reported as the median, with 95% CI. For statistical analysis, 2-tailed Mann-Whitney *U* test was performed. **P < 0.01. (**C**) The indicated NEPC cells were treated with 600 nM SP2509 for 48 hours, and H3K4me2 levels were measured by Western blot analysis. Total histone H3 levels were used as loading controls. (**D**–**I**) LNCaP–N-Myc or MR42D cells stably expressing empty vector, WT *LSD1*, or catalytically inactive mutant *LSD1* (K661A) were transfected with nontargeting control (NTC) or siRNA targeting the 3′ UTR of LSD1. Knockdown of endogenous LSD1 was confirmed with primers specific to the 3′ UTR of endogenous *LSD1* transcript (**D** and **E**). Overexpression of ectopic *LSD1* was confirmed with primers specific to ectopic transcripts (**F** and **G**). Cell viability was measured 96 hours after transfection by cell counting with trypan blue exclusion method (**H** and **I**). *n* = 3. Data are reported as the mean ± SD. For statistical analysis, unpaired 2-tailed Welch’s *t* tests were performed, and *P* values are indicated.

Having established that LSD1 is essential for survival of NEPC cells, we next assessed dose response of pharmacologic LSD1 inhibitors. We used the catalytic inhibitors GSK-LSD1 and GSK-2879552 (20) and an allosteric inhibitor SP2509 (26) — which we previously showed to be effective in blocking LSD1’s noncatalytic function in PRAD cells (13) — in a panel of PRAD and NEPC cell lines (Figure 2A). Interestingly, GSK-LSD1 and GSK-2879552 did not affect cell viability using doses up to 10 μM in any of the prostate cancer cells evaluated, matching prior reports (13, 20) (Supplemental Figure 2A). On the other hand, nearly all the prostate cancer cell lines responded to SP2509 with IC_50_ values in the nanomolar to low micromolar range (Figure 2A). NEPC cell lines were significantly more sensitive to SP2509 than adenocarcinoma cell lines (Figure 2B). We additionally tested SP2577 (seclidemstat), a compound related to SP2509 that recently entered a phase I clinical trial (NCT03600649). SP2577 was also effective in blocking NEPC cell survival, with IC_50_ values in the nanomolar range (Supplemental Figure 2B). We next performed in vitro LSD1 demethylase assays using recombinant LSD1 protein and histone substrates and confirmed that all inhibitors were functionally active (Supplemental Figure 2C).

In addition to demethylating its canonical histone substrate H3 dimethyl lysine 4 (H3K4me2) (27), LSD1 is also known to demethylate nonhistone proteins (28). However, several reports in recent years — including our own — demonstrate that LSD1 regulates important cancer hallmarks independently of its demethylase function (13, 29, 30). Having observed that catalytic LSD1 inhibitors did not affect cell viability (Supplemental Figure 2A), we hypothesized that LSD1 may promote NEPC cell survival independently of its catalytic function. Therefore, we first assessed global H3K4me2 levels in multiple NEPC cell line models after SP2509 treatment and did not observe changes (Figure 2C). Furthermore, to understand the effect of LSD1 inhibition on genome-wide H3K4me2 levels, we examined H3K4me2 by CUT&RUN analysis (31) in LNCaP–N-Myc cells treated with vehicle (DMSO) or SP2509. LSD1 inhibition did not significantly alter H3K4me2 levels, further suggesting that LSD1’s histone demethylase function may not be important for NEPC cell survival (Supplemental Figure 2D). The lack of change in H3K4me2 does not entirely rule out that LSD1’s catalytic function may be essential in NEPC because LSD1 has several nonhistone substrates (28). To understand whether LSD1’s catalytic function was essential for promoting NEPC cell survival, we stably overexpressed WT LSD1 or catalytically inactive mutant LSD1 K661A (32) in 2 different NEPC cell lines. Then, we specifically knocked down endogenous LSD1 using siRNA targeting the 3′ UTR. Knockdown of endogenous LSD1 was confirmed using primers specific to 3′ UTR–expressing endogenous *LSD1* (Figure 2, D and E), while ectopic LSD1 expression was confirmed with primers specific to ectopic *LSD1* mRNA (Figure 2, F and G). Interestingly, both WT and catalytically inactive mutant LSD1 overexpression rescued the viability effects of LSD1 knockdown (Figure 2, H and I). Taken together, these data suggest that LSD1’s catalytic function may not be critical for promoting survival in the NEPC models examined.

Having observed that NEPC cells were susceptible to allosteric LSD1 inhibition, we next sought to determine the cancer hallmarks impacted. LSD1 inhibition induced cell cycle arrest (Supplemental Figure 2, E–H) and eventual cell death (Supplemental Figure 2, I and J). In summary, these data suggest that LSD1 promotes the survival of NEPC cells independently of its catalytic function.

### LSD1 inhibition reactivates the TP53 pathway in NEPC cells.

**Figure 3:**
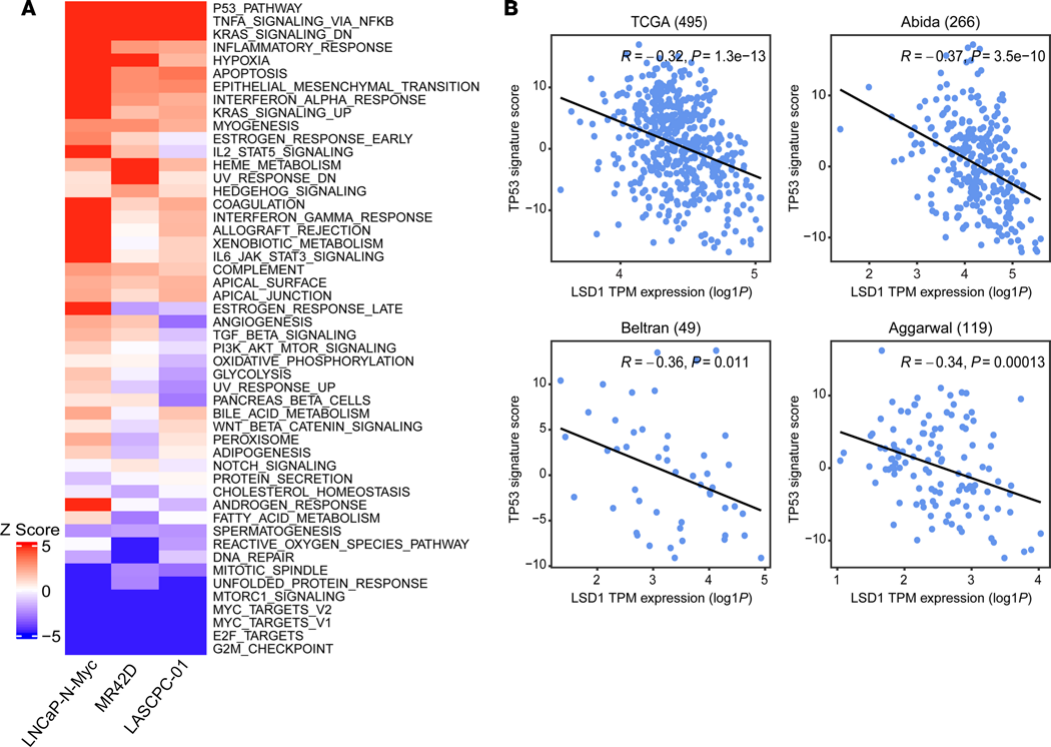
LSD1 inhibition activates the TP53 pathway in NEPC cells. (**A**) Heatmap depicting *Z* scores of the top differentially regulated pathways from RNA-Seq analysis of NEPC cells treated with SP2509. (**B**) Scatter plots and linear fitted lines of TP53 signature score (derived from TP53 activity signature from Chipidza et al.; ref. 8) versus log_1_ *P*-transformed TPM expression of *LSD1* in samples from The Cancer Genome Atlas (TCGA) (35), Abida et al. data set (22), Beltran et al. data set (5), and Aggarwal et al. data set (3). Pearson correlation coefficients (R) and *P* values are shown.

As LSD1 is an important regulator of gene expression (27, 32), we sought to identify key genes and molecular pathways controlled by LSD1 in NEPC. We therefore inhibited LSD1 with SP2509 and performed RNA-Seq in LASCPC-01, LNCaP–N-Myc, and MR42D cell lines. The vast majority of differentially expressed genes after SP2509 treatment were upregulated, suggesting that LSD1 may primarily function as a transcriptional repressor in NEPC (Supplemental Figure 3A). To understand key pathways activated by LSD1 inhibition, we performed hallmark pathway analysis. This demonstrated that *TP53* was the top activated pathway in our NEPC cell line models (Figure 3A). Gene set enrichment analysis (GSEA) also demonstrated significant enrichment of the *TP53* pathway after LSD1 inhibition (Supplemental Figure 3B). Of note, all 3 of these models harbor WT *TP53* alleles (9, 10, 25). Finally, to understand transcriptional regulators whose function changes with LSD1 inhibition, we performed master regulator analysis (33). TP53 was predicted to be the top activated master regulator after LSD1 inhibition in all the NEPC models we examined (Supplemental Figure 3C).

To determine the effect of LSD1 inhibition on the differentiation state of these reprogrammed NEPC cells, we analyzed a previously described luminal differentiation signature (34). We found that LSD1 inhibition induced the activation of a luminal program in both LNCaP–N-Myc and MR42D cells (*P* < 0.1) (Supplemental Figure 3D). The one exception was LASCPC-01, which is derived from basal prostate epithelial cells. The difference in cell of origin may explain why LSD1 inhibition was not sufficient to reactivate a luminal program in LASCPC-01 cells. These data demonstrate that LSD1 inhibition may reactivate a luminal differentiation program in reprogrammed NEPC cells, depending on the cellular context.

We next sought to determine the relationship between *LSD1* expression and TP53 function in patient data sets (3, 5, 22, 35). TP53 activity was measured using an activity signature developed in prostate cancer patient samples by Chipidza et al. (8). There was a significant inverse correlation between *LSD1* expression and TP53 activity in each of the prostate cancer data sets examined (Figure 3B). Taken together, these data suggest that LSD1 is an important regulator of TP53 function in prostate cancer.

### TP53 status modulates the antitumor activity of LSD1 inhibition.

**Figure 4:**
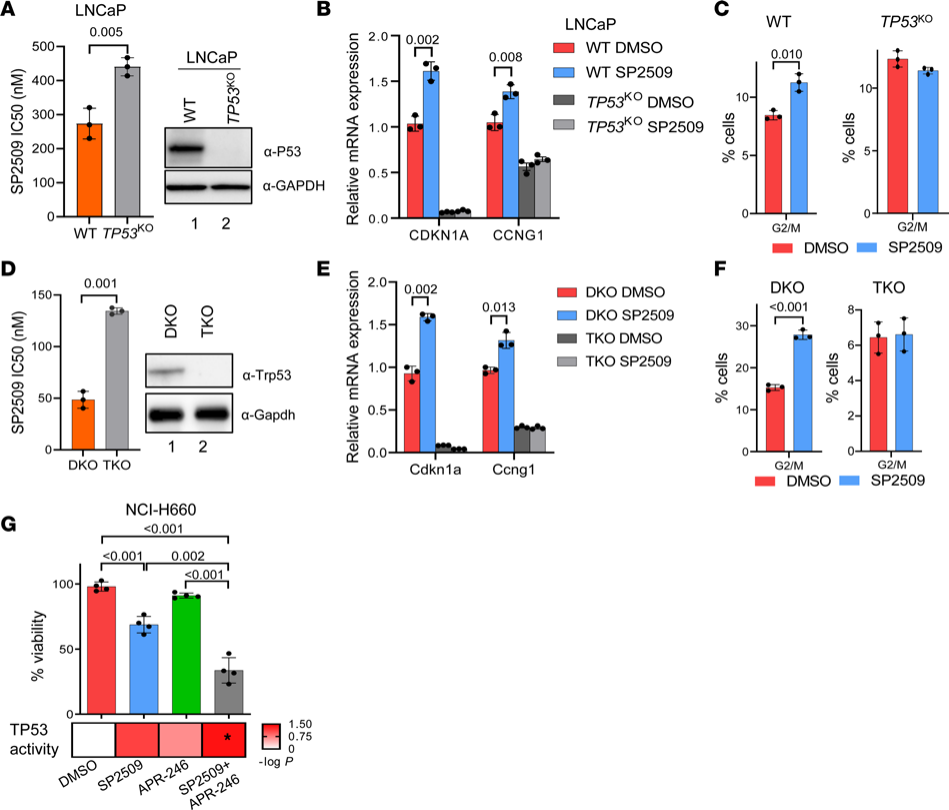
TP53 activation is important for LSD1 inhibition–mediated growth arrest. (**A**) LNCaP cells with WT TP53 or LNCaP cells lacking TP53 expression (LNCaP TP53^KO^) were treated with SP2509. IC_50_ values were calculated (left), *n* = 3. TP53 status of the WT and KO cells were confirmed by measuring TP53 protein levels by Western blotting (right). (**B** and **C**) LNCaP or LNCaP TP53^KO^ cells were treated with 400 nM SP2509 for 48 hours. Expression levels of TP53 target genes (**B**) were measured by qPCR, *n* = 3. Cell cycle profile (**C**) was analyzed by flow cytometry. The percentage of cells in G2/M phase of the cell cycle is shown, *n* = 3. (**D**) Effect of the LSD1 inhibitor SP2509 was tested in mouse prostate cancer cell lines with intact *Trp53* (DKO) or *Trp53* KO (TKO). IC_50_ values were calculated and plotted as bar plot (left), *n* = 3. Trp53 status of the WT and KO cells were confirmed by measuring Trp53 protein levels by Western blotting (right). (**E** and **F**) DKO or TKO cells were treated with 150 nM SP2509 for 48 hours. Expression levels of Trp53 target genes were measured by qPCR, *n* = 3 (**E**). Cell cycle profile was analyzed by flow cytometry. The percentage of cells in G2/M phase of the cell cycle is shown, *n* = 3 (**F**). (**G**) NCI-H660 were treated with 400 nM SP2509 alone, 2 μM APR-246, or the combination for 72 hours. Cell viability was determined by CTG assay (top), *n* = 4. TP53 activity score from single agent or combination treatment was measured by VIPER analysis of RNA-Seq data from NCI-H660 cells upon indicated treatments (bottom). For **A**–**G**, data are reported as the mean ± SD. For statistical analysis, unpaired 2-tailed Welch’s *t* tests were performed, and *P* values are indicated.

It is well established that TP53 functions as a crucial regulator of the G1 and G2 cell cycle checkpoints (36–38). Furthermore, TP53 is known to induce cell cycle arrest and cell death in malignant cells (38, 39). Therefore, we next sought to determine the importance of TP53 reactivation for the cell viability effects we observed with LSD1 inhibition. Using LNCaP parental cells and *TP53*-KO cells we described previously (7), we determined that loss of *TP53* significantly reduced the sensitivity of these cells to LSD1 inhibition, as evidenced by increases in IC_50_ values for SP2509 and SP2577 (Figure 4A and Supplemental Figure 4A). Furthermore, *TP53* KO abrogated the effects of LSD1 inhibition of inducing key TP53 target genes that regulate the cell cycle (e.g., *CDKN1A* and *CCNG1*), demonstrating that TP53 is essential for induction of these genes (Figure 4B). We also analyzed the cell cycle profile after LSD1 inhibition and found *TP53* loss abrogated the cell cycle arrest induced by LSD1 inhibition (Figure 4C and Supplemental Figure 4B).

To confirm these results, we next examined isogenic *TP53*-intact or *TP53*-KO mouse cell line models; the latter exhibit NEPC reprogramming (6). The double KO (DKO) cell line has homozygous loss of *Pten* and *Rb1* while the triple KO (TKO) cell line has loss of *Pten* and *Rb1* in addition to *Trp53* (the mouse *TP53* homolog). Consistent with our results in the LNCaP model (Figure 4, A–C), *Trp53* KO significantly reduced sensitivity to LSD1 inhibition (Figure 4D and Supplemental Figure 4C) and abrogated the induction of the Trp53 target genes *Cdkn1a* and *Ccng1* by LSD1 inhibition (Figure 4E). LSD1 inhibition caused G2/M cell cycle arrest in DKO cells — an effect abrogated by *Trp53* KO in TKO cells (Figure 4F).

To further evaluate the importance of TP53 function for the antitumor effects of LSD1 inhibition, we next examined the cell line NCI-H660, a patient-derived NEPC model that expresses a nonfunctional mutant *TP53*. Similar to the other NEPC cell line models, LSD1 inhibition in NCI-H660 cells reduced cell viability (Figure 4G). Treatment with APR-246, a stabilizer of mutant TP53 that restores WT-like function to mutant TP53 (40), did not significantly change cell viability in these cells. However, combination treatment with SP2509 and APR-246 led to a greater reduction of cell viability than either agent alone (Figure 4G). Furthermore, analysis of the Chipidza TP53 activity score (8) from RNA-Seq data of NCI-H660 cells treated with a single agent (SP2509 or APR-246) or a combination (SP2509 + APR-246) indicated that only the combination treatment significantly activated TP53 function (Figure 4G). These data suggest that stabilization of mutant TP53 protein augments activation of TP53 by LSD1 inhibition. Importantly, this combination effect was not seen with combination treatment in *TP53* WT LASCPC-01 cells (Supplemental Figure 4E), suggesting that the increased efficacy of SP2509 + APR-246 is specific to cells with mutant TP53. Overall, these data suggest that activation of TP53 signaling robustly contributes to the antitumor activity of LSD1 inhibition.

### LSD1 represses TP53 function in cooperation with HDAC2, and LSD1’s catalytic function is dispensable for this effect.

**Figure 5:**
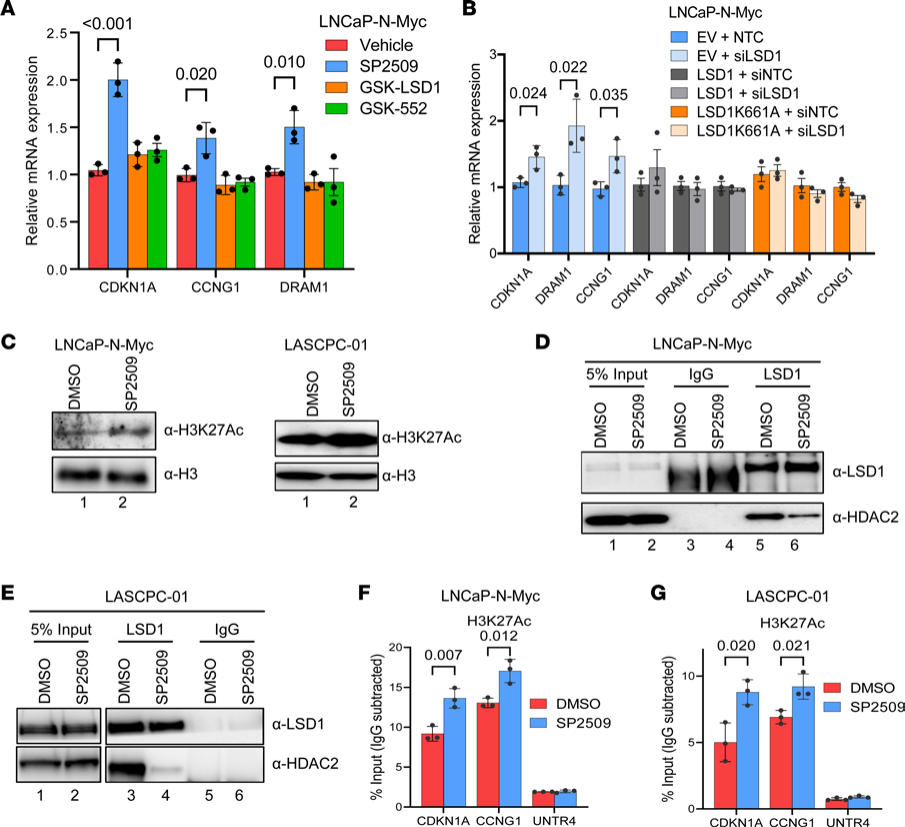
LSD1 inhibition disrupts LSD1-HDAC interaction and increases histone acetylation at TP53 targets. (**A**) LNCaP–N-Myc cells were treated with 1 μM catalytic LSD1 inhibitors (GSK-LSD1 or GSK-552) or 600 nM allosteric LSD1 inhibitor (SP2509), and TP53 target gene expression was measured after 48 hours by qPCR, *n* = 3. (**B**) LNCaP–N-Myc cells stably expressing empty vector, WT *LSD1*, or catalytically inactive mutant *LSD1* (K661A) were transfected with nontargeting control (NTC) or siRNA targeting the 3′ UTR of *LSD1*. The expression of TP53 targets was measured by qPCR, *n* = 3. (**C**) The indicated NEPC cells were treated with 600 nM SP2509 for 48 hours, and H3K27Ac levels were measured by Western blotting. Total histone H3 levels were used as a loading control. (**D** and **E**) The indicated NEPC cells were treated with 600 nM SP2509 for 48 hours, and LSD1-HDAC2 interactions were determined by immunoprecipitation followed by Western blotting. (**F** and **G**) LNCaP–N-Myc (**F**) or LASCPC-01 (**G**) NEPC cells were treated with DMSO vehicle or 600 nM SP2509 for 48 hours. ChIP was performed with anti-H3K27Ac antibodies. qPCR was performed to amplify promoter regions of TP53 targets (*CDKN1A*, *CCNG1*) or a negative control region (UNTR4). Enrichment by IgG control IPs in all the experiments were below 0.1% input, indicating that the enrichment observed with anti-H3K27Ac antibodies in these experiments is specific, *n* = 3. For **A**, **B**, **F**, and **G**, data are reported as the mean ± SD. For statistical analysis, unpaired 2-tailed Student’s *t* tests were performed, and *P* values are indicated.

Next, we sought to probe the mechanism of LSD1-mediated TP53 suppression and NEPC cell survival. To confirm that LSD1’s catalytic function was dispensable for TP53 activation, we performed ChIP experiments for LSD1’s histone demethylation substrate H3K4me2 at the promoters of known TP53 target genes (41) — *CDKN1A*, *CCNG1*, and *DRAM1* — that increased in expression after LSD1 inhibition (Figure 5A). H3K4me2 levels were not altered at these gene promoters (Supplemental Figure 5, A and B). Furthermore, only treatment with SP2509 activated these TP53 target genes, while catalytic LSD1 inhibitors GSK-LSD1 and GSK-552 did not (Figure 5A). Finally, we performed genetic experiments and confirmed that both WT and catalytic-deficient (K661A) LSD1 overexpression abrogated the effects of LSD1 knockdown on inducing TP53 target gene expression (Figure 5B).

LSD1 has been shown to regulate TP53 function by demethylating TP53 at K370 (42). To test whether LSD1 regulates TP53 in a demethylation-independent manner, we reconstituted TP53-null cells with either WT or demethylation-deficient (K370R) mutant TP53 (43) and examined TP53 activation by SP2509. Overexpression of either WT or K370R mutant TP53 activated TP53 target genes upon LSD1 inhibition (Supplemental Figure 5, C and D). These data suggest that LSD1 regulates TP53 function independently of demethylation. Taken together, these data suggest that LSD1 represses TP53 function independently of its catalytic function in NEPC.

LSD1 is a member of several repressive complexes and has been shown to cooperate with histone deacetylases, including HDAC2, to repress gene expression (27). To further understand the mechanisms by which LSD1 inhibition reactivates gene expression, we measured global levels of the activating histone mark H3 lysine 27 acetylation (H3K27Ac) by Western blot. H3K27Ac levels were increased with LSD1 inhibition (Figure 5C). We next tested whether LSD1 inhibition disrupted interaction of LSD1 with HDAC2 by performing immunoprecipitation followed by Western blot (IP-WB). SP2509 treatment blocked LSD1-HDAC2 interactions in multiple NEPC cell lines (Figure 5, D and E).

We next examined levels of H3K27Ac at TP53 target gene promoters after LSD1 inhibition. LSD1 inhibition increased H3K27Ac at both *CDKN1A* and *CCNG1* promoters (Figure 5, F and G). Since we observed that LSD1 inhibition disrupted the LSD1-HDAC2 complex, we next tested whether HDAC inhibition recapitulated the effects of LSD1 inhibitor treatment on TP53 activation. Treatment with the HDAC inhibitor Trichostatin A (TSA) reactivated TP53 target genes in NEPC cell lines (Supplemental Figure 5, E and F). Taken together, these data suggest that LSD1 associates with HDAC2 to repress TP53 signaling in NEPC and that LSD1’s catalytic function is dispensable for this effect.

**Figure 6:**
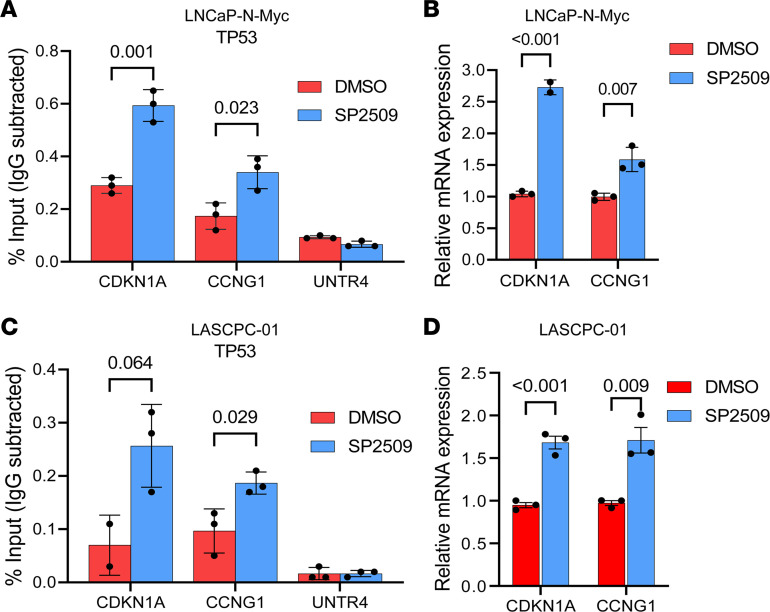
LSD1 inhibition increases TP53 occupancy at chromatin. (**A**) LNCaP–N-Myc cells were treated with DMSO vehicle or 600 nM SP2509 for 48 hours. ChIP was performed with anti-TP53 antibodies. qPCR was performed to amplify promoter regions of TP53 targets (*CDKN1A*, *CCNG1*) or a negative control region (UNTR4), *n* = 3. (**B**) LNCaP–N-Myc cells were treated with DMSO vehicle or 600 nM SP2509 for 48 hours. Expression of TP53 targets was analyzed by qPCR, *n* = 3. (**C**) LASCPC-01 cells were treated with DMSO vehicle or 600 nM SP2509 for 48 hours. ChIP was performed with anti-TP53 antibodies. qPCR was performed to amplify promoter regions of TP53 targets (CDKN1A, CCNG1) or a negative control region (UNTR4), *n* = 3. (**D**) LASCPC-01 cells were treated with DMSO vehicle or 600 nM SP2509 for 48 hours. Expression of TP53 targets were analyzed by qPCR, *n* = 3. For **A** and **C**, anti-IgG antibodies were used in each ChIP experiment to determine nonspecific pull-down. Enrichment by IgG pull-down in all the experiments was below 0.1% input, indicating that the enrichment observed with anti-TP53 antibodies in these experiments is specific. For **A**–**D**, data are reported as the mean ± SD. For statistical analysis, unpaired 2-tailed Student’s *t* tests were performed, and *P* values are indicated.

Having observed increases in H3K27Ac at TP53 target promoters, we hypothesized that LSD1 inhibition may activate the TP53 pathway by also regulating TP53 occupancy. We confirmed that LSD1 inhibition increased TP53 binding at its target genes (Figure 6, A and C, and Supplemental Figure 6A), coinciding with induction of these genes’ expression (Figure 6, B and D, and Supplemental Figure 6B). These data suggest that LSD1 inhibition reactivates TP53 signaling by also enhancing TP53 occupancy at its target genes.

### LSD1 suppression inhibits NEPC tumor growth in vivo.

**Figure 7:**
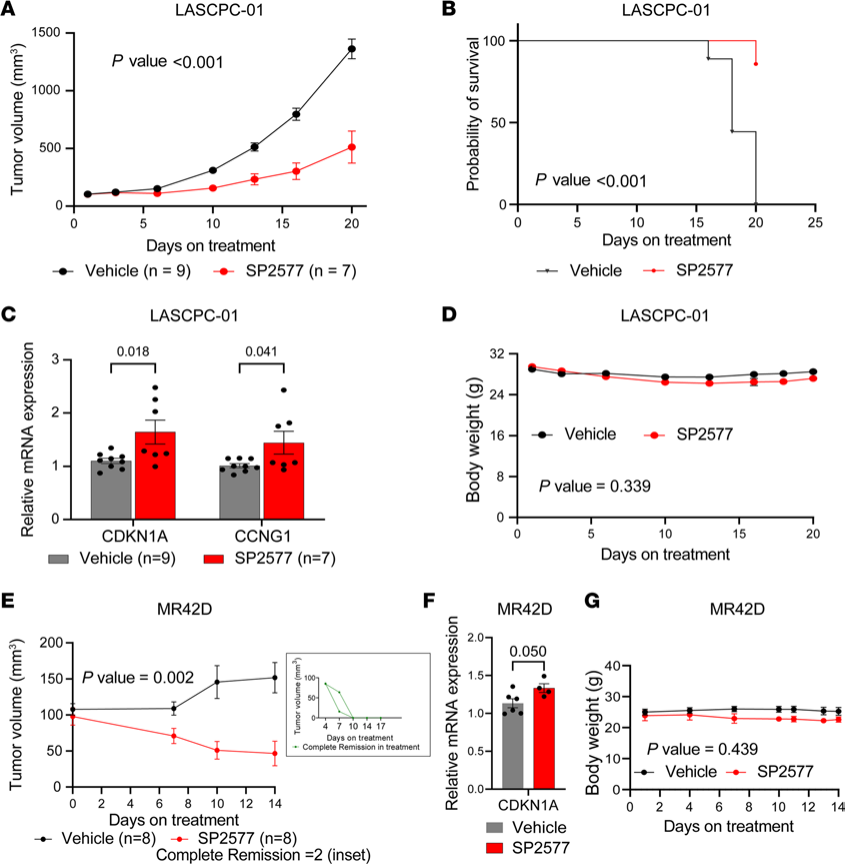
LSD1 inhibition suppresses NEPC tumor growth in vivo. (**A**) Tumor growth of LASCPC-01 xenografts treated with vehicle or SP2577 was measured as a function of time. Data are presented as mean ± SEM. For statistical analysis, a mixed-effects model 2-way ANOVA was performed, and the *P* value is indicated. (**B**) Survival analysis was performed for mice implanted with LASCPC-01 xenografts using the time-to-tumor volume of 1,000 mm^3^ as a surrogate for survival and presented as Kaplan-Meier curve. For statistical analysis, a log-rank *P* value was calculated and is indicated. (**C**) Expression of TP53 target gene *CDKN1A* was analyzed by qPCR in tumors harvested at endpoint. Data are presented as mean ± SD. For statistical analysis, unpaired 2-tailed Student’s *t* tests were performed, and *P* values are indicated. (**D**) Body weight of mice during treatment was measured as a function of time. Data are presented as mean ± SEM. For statistical analysis, a mixed-effects model 2-way ANOVA was performed, and the *P* value is indicated. (**E**) Tumor volume of MR42D xenografts was measured as a function of time. The inset shows 2 tumors that underwent complete remission after SP2577 treatment. Data are presented as mean ± SEM. For statistical analysis, a mixed-effects model 2-way ANOVA was performed, and the *P* value is indicated. (**F**) Expression of the TP53 target gene *CDKN1A* was analyzed by qPCR in tumors harvested at the endpoint. Data are presented as mean ± SD. For statistical analysis, an unpaired Student’s *t* test was performed, and the *P* value is shown. (**G**) Body weight of mice during treatment was measured as a function of time and plotted. Data are presented as mean ± SEM. For statistical analysis, a mixed-effects model 2-way ANOVA was performed, and the *P* value is indicated.

Because we observed growth suppressive effects of LSD1 inhibitor treatment in vitro, we next sought to determine the antitumor activity of LSD1 suppression in vivo. For these experiments, we elected to use SP2577 because of its greater solubility than SP2509 and because SP2577 has recently entered clinical trials (NCT03600649). SP2577 treatment suppressed growth of LASCPC-01 xenografts (Figure 7A). SP2577 also improved survival of LASCPC-01 tumor–bearing mice (Figure 7B). Analysis of the harvested tumors revealed activation of TP53 targets genes, suggesting that TP53 activation may contribute to the growth suppression observed (Figure 7C). Of note, body weights of mice treated with SP2577 were similar to mice treated with vehicle (Figure 7D), suggesting that SP2577 is well tolerated and indicating the potential safety of SP2577 treatment for NEPC.

We also determined the effect of LSD1 knockdown on growth of LASCPC-01 tumors implanted in mice using a doxycycline-inducible shRNA. Doxycycline diet–fed mice had significantly smaller tumors versus control mice (Supplemental Figure 7A). We measured LSD1 expression in mice fed with doxycycline diets and confirmed that the growth suppressive effects were linked to LSD1 protein suppression (Supplemental Figure 7B). The TP53 target p21 (the protein encoded by the *CDKN1A* gene) was higher in LSD1-knockdown tumors, suggesting that TP53 activation may contribute to the observed antitumor effect (Supplemental Figure 7B). There were no significant differences in body weight between groups (Supplemental Figure 7C).

Finally, we sought to test LSD1 inhibition in a t-NEPC model generated after long-term treatment with the ARSI enzalutamide. Therefore, we implanted MR42D cells in castrated mice and treated them with enzalutamide alone or SP2577 + enzalutamide. SP2577 treatment suppressed tumor growth (Figure 7E). Importantly, we observed complete remission in 2 of the 8 tumors in the SP2577 group (Figure 7E, inset), highlighting the potential of SP2577 for blocking growth of t-NEPC. Analysis of the harvested tumors indicated that *CDKN1A* was upregulated, suggesting that TP53 activation may contribute to the antitumor activity (Figure 7F). Mouse body weights were similar, demonstrating that treatment was well tolerated (Figure 7G). Taken together, these data further demonstrate that LSD1 is important for NEPC tumor growth and suggest that LSD1 inhibition is worthy of further study in patients with NEPC.

## Discussion

NEPC is increasingly recognized as an important subtype of prostate cancer associated with tumor aggressiveness and lethality (3, 5). There are several reprogramming factors whose loss or gain of function contributes to NEPC lineage plasticity (5, 6, 9–12). However, there are still no effective treatments for NEPC, demonstrating a compelling need to understand the biology of these tumors more deeply so we may identify new targetable factors. Recent studies highlight the importance of epigenetic dysregulation in prostate cancer and targeting epigenetic factors in this disease (44, 45). LSD1 is a key epigenetic regulator that promotes stemness and therapy resistance in multiple cancers (14–17, 28, 29, 46). Our results herein demonstrate that LSD1 promotes the survival of tumors that have undergone NEPC reprogramming and does so through noncanonical, demethylase-independent mechanisms that suppress TP53 activation.

Importantly, we found that NEPC cell lines were more sensitive to LSD1 inhibition than adenocarcinoma cells. Progression of AR-dependent adenocarcinoma through lineage plasticity to AR-independent forms of prostate cancer, such as double-negative prostate cancer (DNPC) — lacking both AR expression and NEPC differentiation — or NEPC through lineage plasticity, is now increasingly recognized (5, 11, 12, 24, 47, 48). LSD1 has been shown to promote therapy resistance in multiple cancers (28, 29, 46). Specifically in prostate cancer, our previous work revealed that LSD1 promotes AR-independent survival of both adenocarcinoma and DNPC cells (13). Our results demonstrate higher expression of LSD1 in NEPC and increased sensitivity of NEPC cells to LSD1 inhibition. Thus, LSD1 appears to be a target of importance in NEPC — a particularly virulent AR-independent subtype — and our results suggest that suppression of TP53 function may contribute to LSD1’s effects in NEPC.

TP53 is a critical tumor suppressor commonly altered in NEPC (7). TP53 restrains lineage plasticity by blocking the function of specific master regulator transcription factors (49). Loss of *TP53* has been shown to cooperate with loss of other tumor suppressors such as *PTEN* and *RB1* to promote lineage plasticity (6, 7). While genetic loss of *TP53* occurs in 33% of prostate cancers, a recent report demonstrated that 17% of prostate cancers harbor WT TP53 alleles but exhibit loss of TP53 transcriptional function (8). Our results suggest that LSD1 may be a critical negative regulator of TP53 in NEPC. Thus, LSD1 inhibition may provide therapeutic benefit by reactivating TP53 function in tumors with nongenomic loss of TP53. In addition, 20% of NEPC tumors have been shown to harbor a mutant *TP53* allele (5). Our data suggest that combining mutant TP53 stabilizers — that are now in clinical trials — with LSD1 inhibition is a promising strategy in these tumors to reactivate TP53 protein function. These results may have implications beyond NEPC, as TP53 plays a crucial role in restraining PRAD cancer cells from acquiring a mesenchymal, stem-like phenotype that is associated with ARSI resistance and poor clinical outcome (4, 50, 51).

There are multiple control points for regulating TP53 function, including tightly regulated posttranslational modifications and interactions with coactivators in response to cellular stimuli (52). Phosphorylation at key residues in response to genotoxic stress or DNA damage regulates TP53 function (52). Of note, a prior report in breast cancer also described the importance of TP53 protein methylation and demonstrated that LSD1 demethylates TP53, affecting its function and protein stability (42). However, our data using genetic and pharmacologic suppression of LSD1 strongly suggest that LSD1’s catalytic function is dispensable for repressing TP53 signaling and promoting survival of NEPC cells. Data from the rescue experiment with a demethylation-deficient mutant TP53 further support a demethylation-independent mechanism of TP53 repression by LSD1. Rather, our data indicate that LSD1 may repress TP53 function through regulation of histone acetylation and TP53 occupancy at its target gene promoters. In line with previous studies (53), it is possible that the cellular context or lineage of a cell may impact mechanisms by which LSD1 functions. In prostate cancer, we hypothesize that LSD1-mediated repression of TP53 function may enable prostate cancer cells to acquire therapy resistance and lose AR dependence.

It is now clear that chromatin modifying enzymes have both canonical enzymatic functions and noncanonical functions and work in concert with other factors (13, 30, 42). LSD1 is no exception. Importantly, LSD1 inhibition disrupts interactions with key complex members (13, 28, 29, 46). Herein, we determined that an LSD1-HDAC2 complex suppresses TP53 signaling to promote NEPC cell survival. Finally, suppression of LSD1 by RNAi or allosteric inhibition in vivo suppressed NEPC tumor growth, demonstrating the importance of targeting noncanonical functions of LSD1 in NEPC. These data suggest that further investigation of LSD1’s scaffold function may be warranted to identify additional control points or interaction partners whose suppression blocks NEPC cell survival — work we are currently undertaking.

While the majority of pathways after LSD1 inhibition were upregulated, our data also demonstrate downregulation of key proliferative pathways (e.g., E2F1, G2M checkpoint, Myc) across NEPC models. Indeed, prior work in PRAD models suggests that LSD1 and E2F1 cooperate to promote cell survival (54, 55). E2F1 is known to play a key role in lineage plasticity during prostate cancer progression (11). Thus, it is possible that LSD1 also promotes NEPC cell survival by cooperating with E2F1.

In summary, we determined that LSD1 promotes NEPC cell survival by repressing the function of the tumor suppressor TP53 and that LSD1 does so independently of its catalytic function. The fact that pharmacologic inhibition of LSD1 suppressed NEPC tumor growth in vivo and was well tolerated suggests that LSD1 inhibition is a promising treatment direction for NEPC.

## Methods

Further information can be found in Supplemental Methods.

### Cell lines.

LNCaP-P53KO (7) and LASCPC-01 (10) were described previously. MR42D (gift from A. Zoubeidi, University of British Columbia, Vancouver, British Columbia, Canada), LNCaP–N-Myc (gift from D. Rickman, Weill Cornell Medicine, New York, New York, USA), and DKO and TKO cells (gifts from L. Ellis, Cedars-Sinai Medical Center, Los Angeles, California, USA) were cultured as described previously (6, 7, 9–11, 56). LNCaP (CRL-1740) and NCI-H660 cells (CRL-5813) were purchased from ATCC and cultured according to their recommendation. All cell lines were validated with STR DNA fingerprinting by Genetica Cell Line Testing (a LabCorp brand) and regularly tested for Mycoplasma contamination by the MycoAlert Mycoplasma Detection Kit (Lonza, LT07-318).

### Chemicals.

SP2577 (seclidemstat) (catalog HY-103713A), trichostatin-A (catalog HY-15144), and enzalutamide (catalog HY-70002) were obtained from MedChemExpress. SP2509 (catalog S7680) and APR-246 (eprenetapopt, also referred to as PRIMA-1MET) (catalog S7724) were obtained from Selleck Chemicals. All the drugs were dissolved in DMSO, and DMSO was used as vehicle control for drug-treatment assays. Doxycycline hyclate (MilliporeSigma, catalog D9891) dissolved in water was used for experiments with doxycycline-inducible constructs at a final concentration of 500 ng/mL.

### Antibodies.

Anti-LSD1 (Cell Signaling Technology, catalog 2139), anti-TP53 (Santa Cruz Biotechnology, catalog sc-126), anti-p21(CDKN1A) (Cell Signaling Technology, catalog 2947), anti-GAPDH (Santa Cruz Biotechnology, catalog sc-32233), anti-H3K4me2 (Cell Signaling Technology, catalog 9725), anti-H3K27Ac (Active Motif, catalog 39133), and anti–Histone H3 (MilliporeSigma, catalog 06-755) were used for protein detection by Western blotting.

### Statistics.

GraphPad Prism version 9.4.1 was used for statistical analysis and plotting graphs. The data from 3 biological replicates are presented as mean ± SD, and SD represents the deviation between the biological replicates. Comparisons between treatment and control groups for cell line viability experiments with siRNA and with drug treatment, quantitative PCR (qPCR) expression, and ChIP-qPCR experiments used 2-tailed unpaired Student’s *t* tests, and a *P* value less than 0.05 was considered significant. For comparison of IC_50_ values between adenocarcinoma and NEPC/reprogrammed models, a 2-tailed Mann-Whitney *U* test was used. A *P* value less than 0.05 was considered significant. For gene signature analyses, a *P* value less than 0.1 was considered significant.

### Study approval.

All the animal studies were performed under the animal protocols (PRO00009620 and PRO00009560), reviewed, and approved by the Institutional Animal Care and Use Committee (IACUC) at the University of Michigan.

### Data availability statement.

RNA-Seq data and corresponding clinical annotations of tumor samples are available through the following: the Beltran et al. data set (5), the Aggarwal data set from West Coast Dream Team (WCDT) (3, 57), and the Abida data set (22). The gene expression data of primary PRAD samples were obtained from The Cancer Genome Atlas (TCGA; https://www.cancer.gov/tcga) via TCGAbiolinks R/Bioconductor package (version 2.20.1) (58). The RNA-Seq data sets reported in this article are deposited into NCBI Gene Expression Omnibus (GEO) (GSE218993). The CUT&RUN data sets reported in this article are deposited into NCBI Gene Expression Omnibus (GEO) (GSE235211). All raw data are available in the Supplemental Data Values. See complete unedited blots in the supplemental material.

## Author contributions

JJA and AK designed the research; AK, ZD, DF, CZ, AS, OAS, ER, WKS, and JAK performed experiments; AK, ZD, and DF acquired data; RM, XMW, AU, and AMC performed IHC evaluation; YMH, KB, AB, VR, IC, and AR performed computational analyses; AK, ZD, DF, JAY, and JJA analyzed the data; JKL and PSN provided cell line models; CM provided patient tumor samples; AK, ZD, JAY, and JJA wrote the manuscript; JKL, CM, PSN, ZX, JAY, and JJA revised the manuscript. All authors read and approved the final manuscript.

## Supplementary Material

Supplemental data

Supporting data values

## Version 1 07/13/2023

In-Press Preview

## Version 2 08/08/2023

Electronic publication
