# Supplementary material for: LSD1 promotes prostate cancer reprogramming by repressing TP53 signaling independently of its demethylase function
Source: JCI Insight. 2023 Aug 8;8(15):e167440. doi: 10.1172/jci.insight.167440 (PMC10445684; doi:10.1172/jci.insight.167440)
Supplement: Supplemental data [file jciinsight-8-167440-s127.pdf]

LSD1 Promotes Prostate Cancer Cell Reprogramming by Repressing TP53 Signaling Independently of Its Demethylase Function

Anbarasu Kumaraswamy[1,2], Zhi Duan[1,2], Diana Flores[1,2], Chao Zhang[1,2], Archana Sehrawat[3], Ya-Mei Hu[3,4], Olivia A. Swaim[1,2,5], Eva Rodansky[1,2], William K. Storck[1,2], Joshua A. Kuleape[1,2], Karan Bedi[2,6], Rahul Mannan[7], Xiao-Ming Wang[7,8], Aaron Udager[7], Visweswaran Ravikumar[9], Armand Bankhead III[1,9], Ilsa Coleman[10], John K. Lee[10], Colm Morrissey[11], Peter S. Nelson[10], Arul M. Chinnaiyan[2,6,7,12,13], Arvind Rao[2,8,14,15], Zheng Xia[3,4], Joel A. Yates[1,2], Joshi J. Alumkal[1,2,8]

[1] Department of Internal Medicine, University of Michigan, Ann Arbor, MI, USA
[2] Rogel Cancer Center, University of Michigan, Ann Arbor, MI, USA
[3] Knight Cancer Institute, Oregon Health & Science University (OHSU), Portland, OR, USA
[4] Biomedical Engineering Department, Oregon Health & Science University (OHSU), Portland, OR, USA
[5] College of Literature, Science, and the Arts, University of Michigan, Ann Arbor, MI, USA
[6] Department of Biostatistics, School of Public Health, University of Michigan, Ann Arbor, MI, USA
[7] Department of Pathology, University of Michigan Medical School, Ann Arbor, MI, USA
[8] Michigan Center for Translational Pathology, Ann Arbor, MI, USA
[9] Department of Computational Medicine & Bioinformatics, University of Michigan, Ann Arbor, MI, USA
[10] Divisions of Human Biology and Clinical Research, Fred Hutchinson Cancer Research Center, Seattle, WA, USA
[11] Department of Urology, University of Washington, Seattle, WA, USA
[12] Department of Urology, University of Michigan Medical School, Ann Arbor, MI, USA
[13] Howard Hughes Medical Institute, Ann Arbor, MI, USA
[14] Department of Radiation Oncology, University of Michigan, Ann Arbor, MI, USA
[15] Department of Biomedical Engineering, University of Michigan, Ann Arbor, MI, USA

Corresponding author:
Joshi J. Alumkal, 7312 Rogel Cancer Center, SPC 5948, 1500 East Medical Center Drive, Ann Arbor, MI 48109. Phone: +1-(734) 936-9868. E-mail: jalumkal@med.umich.edu

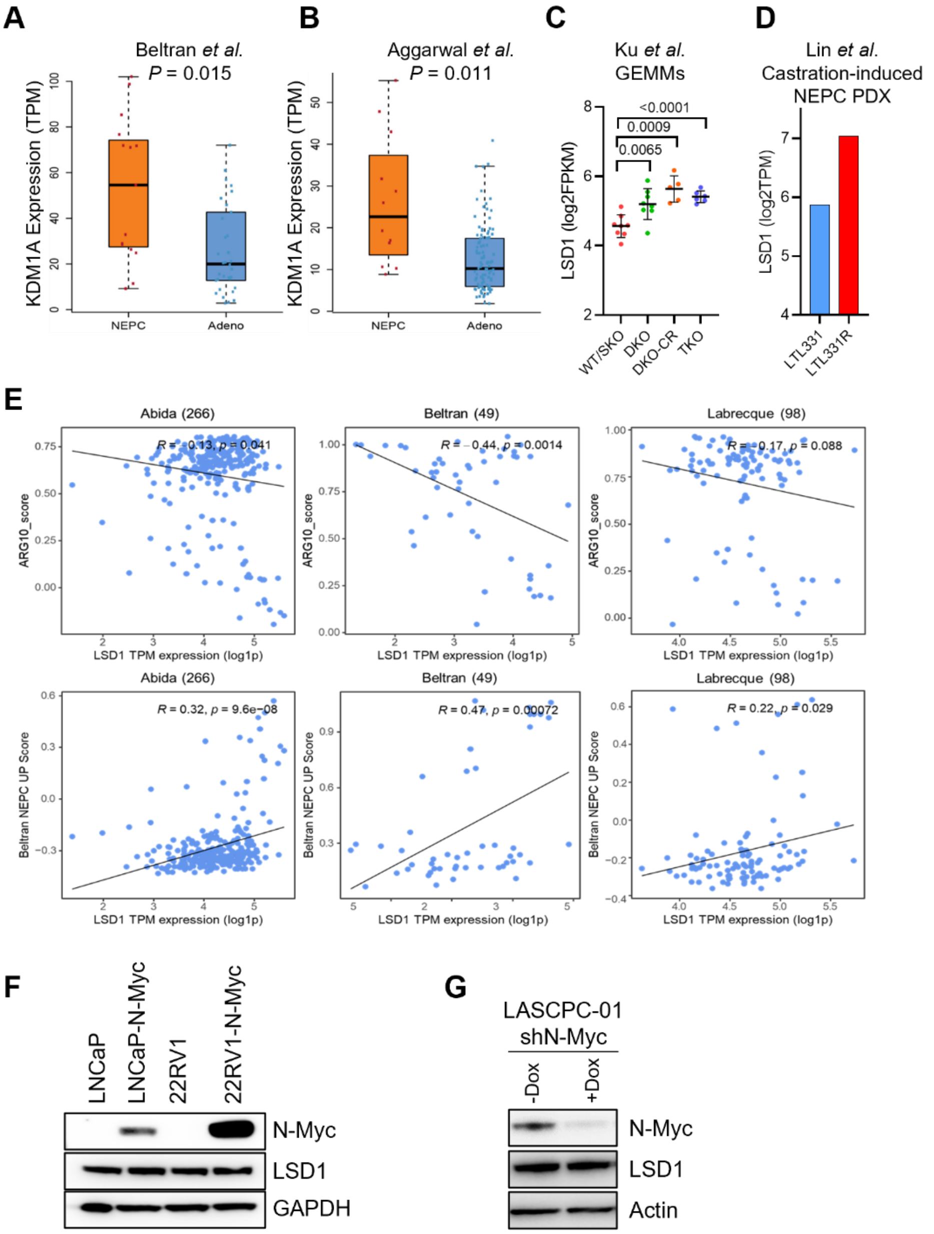

Supplemental Figure 1: *LSD1* is upregulated in NEPC tumors. *(Related to Figure 1)* (**A-B**) *LSD1 (KDM1A)* mRNA levels were quantified in neuroendocrine (NEPC) vs. adenocarcinoma (Adeno) prostate cancer samples from Beltran *et al.* (**A**) or Aggarwal *et al.* (**B**). For statistical analysis, unpaired two-tailed Student's t-tests were performed, and *P* values are indicated. (**C-D**) *LSD1* mRNA levels were quantified in tumors of *Pten/Rb1/Trp53* knock out genetically-engineered mouse models SKO (*Pten*$^{-/-}$), DKO (*Pten*$^{-/-}$ *Rb1*$^{-/-}$), DKO-CR (castrate-resistant DKO tumors), and TKO (*Pten*$^{-/-}$ *Rb1*$^{-/-}$ *Trp53*$^{-/-}$) developed by Ku, *et al*. (**C**) and a PDX model of castration-induced NEPC transdifferentiation developed by Lin, *et al.* (**D**). For (**C**) statistical analysis, an unpaired two-tailed Welch's t-test was performed, and *P* values are indicated. (**E**) Scatter plots and linear fit lines of AR function (ARG10 score) or NEPC signature (Beltran NEPC Up score) vs. log1p-transformed TPM expression of *LSD1* in samples from the Abida, *et al*., Beltran, *et al*., and Labrecque, *et al*. datasets. Pearson correlation coefficients (R) and *P* values are shown. (**F**) Parental or stable N-Myc overexpressing LNCaP and 22Rv1 cells were analyzed for LSD1 protein expression by Western blotting. (**G**) LASCPC-01 cells stably expressing Dox inducible shN-Myc were cultured with or without dox (500 ng/mL) for 96 hours and LSD1 levels were measured by Western blotting.

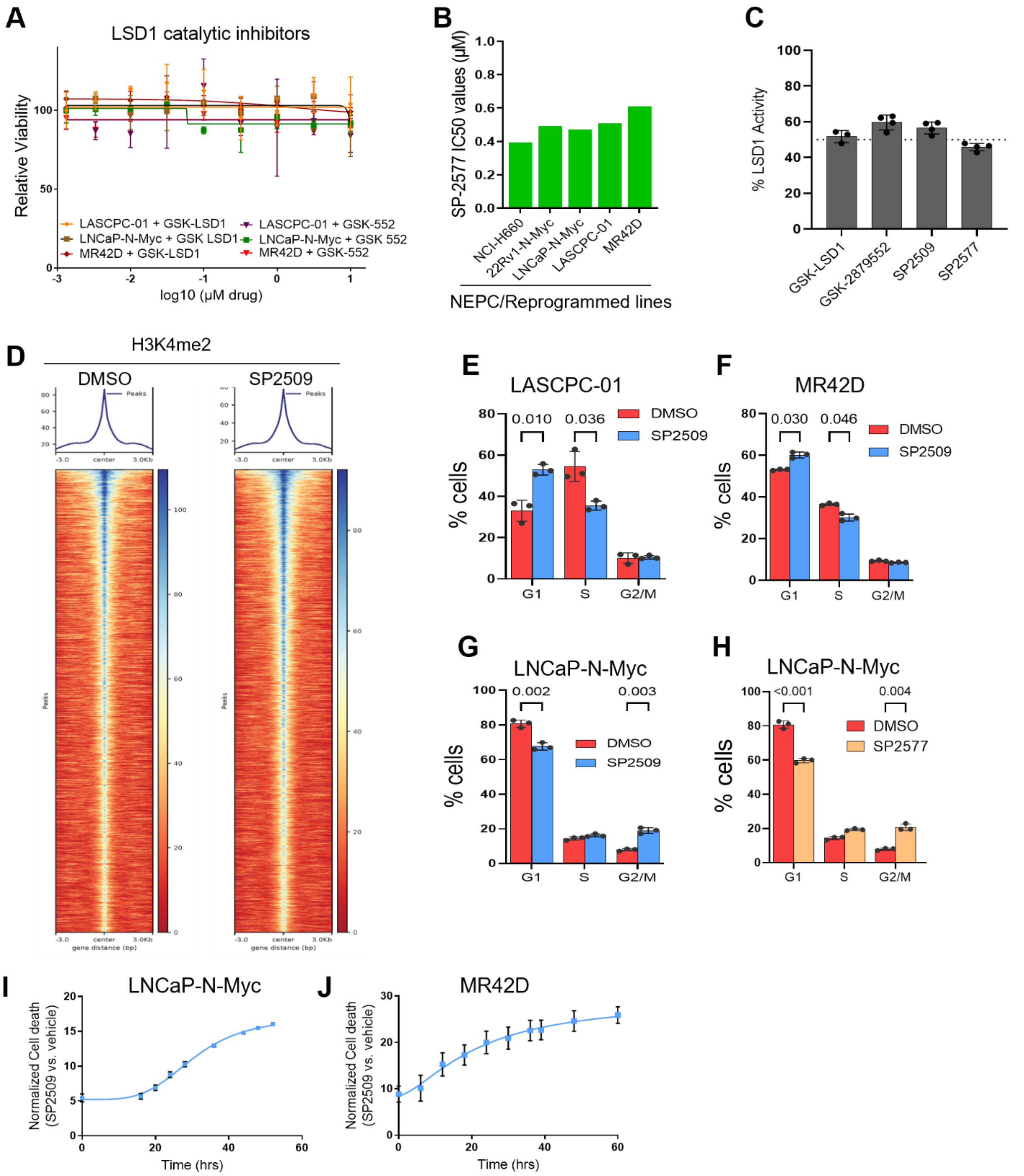

Supplemental Figure 2: NEPC cells are susceptible to allosteric LSD1 inhibition. *(Related to Figure 2)* (**A**) Dose response curves using the LSD1 catalytic inhibitors GSK-LSD1 and GSK-552 in LASCPC-01, LNCaP-N-Myc, and MR42D cell lines treated for 72 hours. (**B**) SP2577 (Seclidemstat) was tested in a panel of NEPC cell lines for 72 hours. IC_{50} values were calculated from the dose-response viability curve. (**C**) LSD1 demethylase assays with recombinant LSD1 and histone H3 peptide (methylated at lysine 4) were performed in the presence of the different LSD1 inhibitors: GSK-LSD1, GSK2879552, SP2509, and SP2577. The percent of LSD1 activity was calculated relative to the maximum LSD1 activity set as 100% in the DMSO control-treated sample. (**D**) Heatmaps depicting genome-wide distribution of H3K4me2 measured by CUT&RUN in LNCaP-N-Myc cells treated with DMSO vehicle or SP2509 for 48 hours. (**E-G**) LASCPC-01 (**E**),

MR42D (**F**), or LNCaP-N-Myc (**G**) cells were treated with 600 nM SP2509 for 48 hours. Cell cycle profiles were measured by propidium iodide staining followed by flow cytometry analysis. Cell cycle profiles were fitted, and the percentage of cells in each phase of the cell cycle was calculated using FlowJo software, n=3. (**H**) LNCaP-N-Myc cells were treated with 600 nM SP2577 for 48 hours. The cell cycle profile was analyzed as indicated in (**G**), n=3. (**I-J**) LNCaP-N-Myc (**I**) or MR42D (**J**) cells were treated with vehicle or 600 nM SP2509, and cell death was measured at the indicated time points. Cell death levels were normalized to vehicle-treated control cells, n=3. For (**A-C** and **E-J**) data are reported as the mean ± SD. For (**A-C** and **E-H**) statistical analysis, unpaired two-tailed Welch's t-test was performed, and *P* values are indicated.

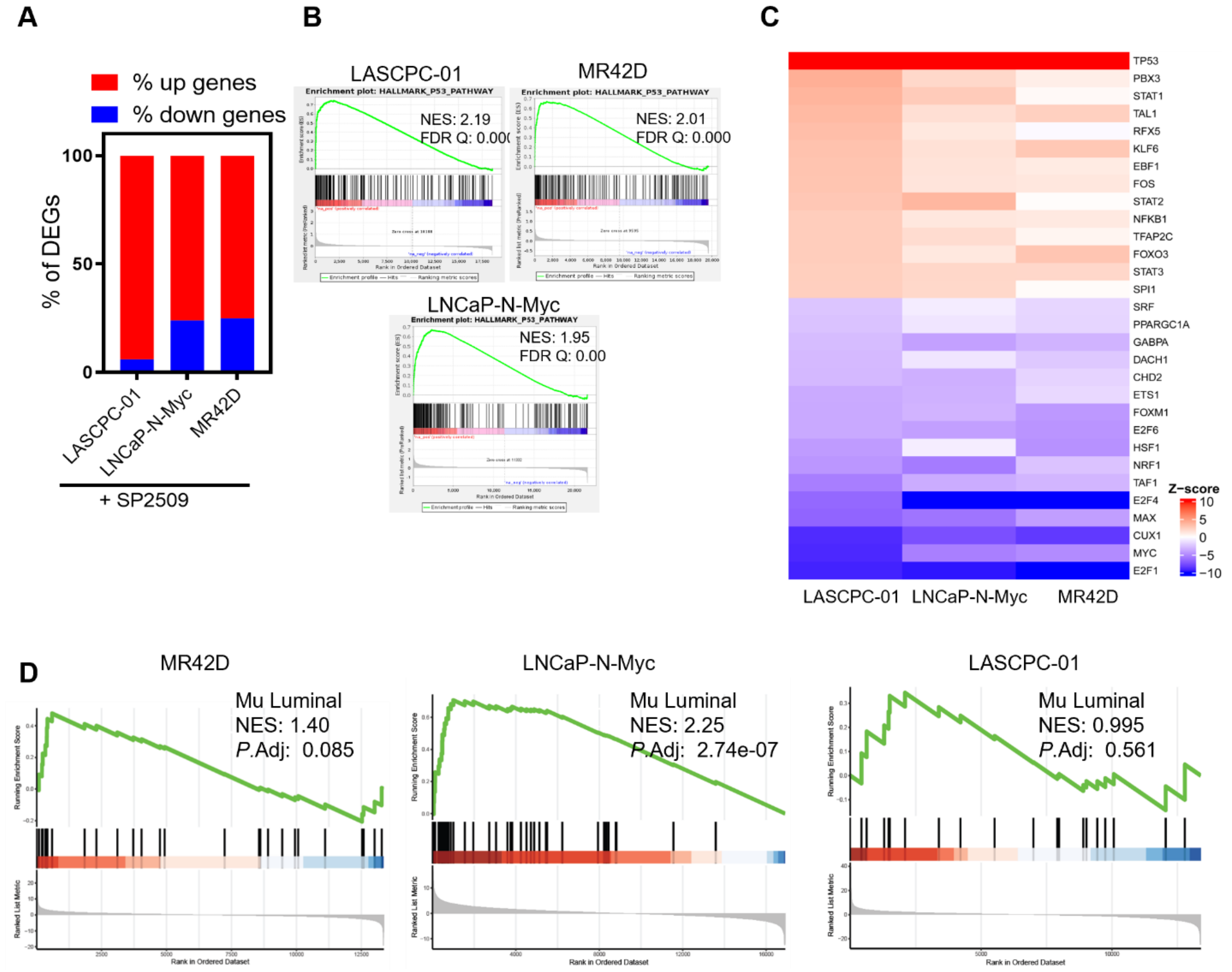

Supplemental Figure 3: LSD1 represses TP53 function and luminal differentiation. *(Related to Figure 3)* (**A**) Bar plots indicating percentage of differentially up- or downregulated genes from RNA-seq analysis after 48 hour SP2509 treatment in the indicated NEPC cell lines. (**B**) Gene-set enrichment analysis plot showing the TP53 pathway signature from RNA-seq after SP2509 treatment of the indicated NEPC cell lines. (**C**) Heatmap depicting z-scores of master regulators from RNA-seq analysis data differentially regulated after SP2509 treatment of the indicated NEPC cell lines. (**D**) Gene-set enrichment analysis plots show luminal differentiation signature in indicated NEPC cell lines upon SP2509 treatment.

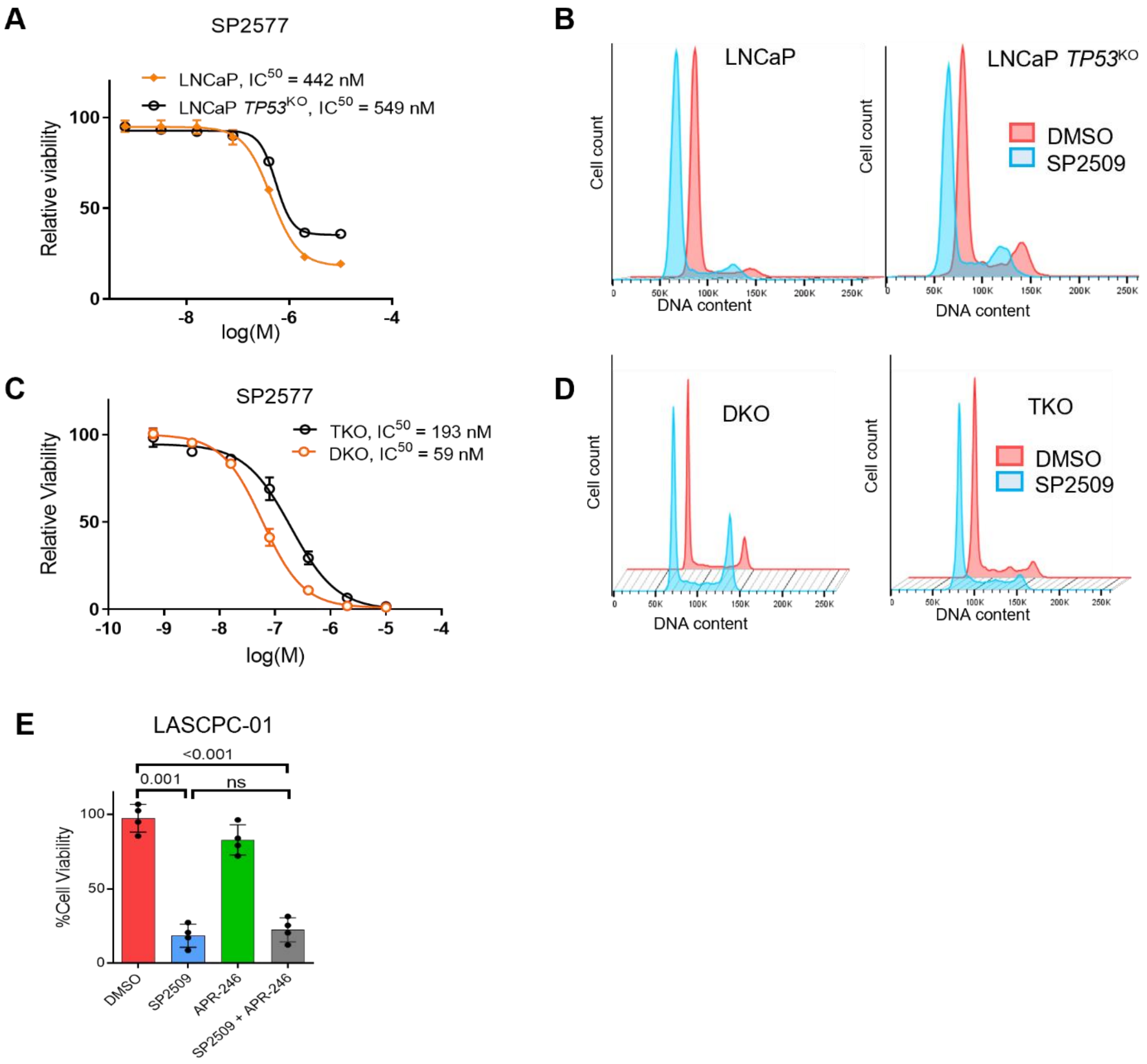

Supplemental Figure 4: Reactivation of the TP53 pathway by LSD1 inhibition reduces NEPC cell survival. *(Related to Figure 4)* (**A**) LNCaP cells with wild-type TP53 or LNCaP cells lacking TP53 (LNCaP $TP53^{KO}$) were treated with SP2577 for 72 hours. IC_{50} values were calculated from the dose-response viability curves, n=4. (**B**) Cell cycle profiles for indicated cell lines after 400 nM SP2509 treatment for 48 hours are depicted as staggered histograms. (**C**) Mouse prostate cancer cell lines with intact Trp53 (DKO) or Trp53 knock-out (TKO) were treated with SP2577 for 72 hours. IC_{50} values were calculated from the dose-response viability curves, n=4. (**D**) Cell cycle profiles for indicated cell lines after 150 nM SP2509 treatment for 48 hours are depicted as staggered histograms. (**E**) 600 nM SP2509 alone or in combination with the TP53 mutant stabilizer (2 µM APR-246) for 72 hours was tested in LASCPC-01 harboring wild-type *TP53* alleles, n=3. Unpaired two-tailed Welch's t-tests were performed, and *P* values are indicated. For (**A**, **C**, and **E**), data are reported as the mean ± SD.

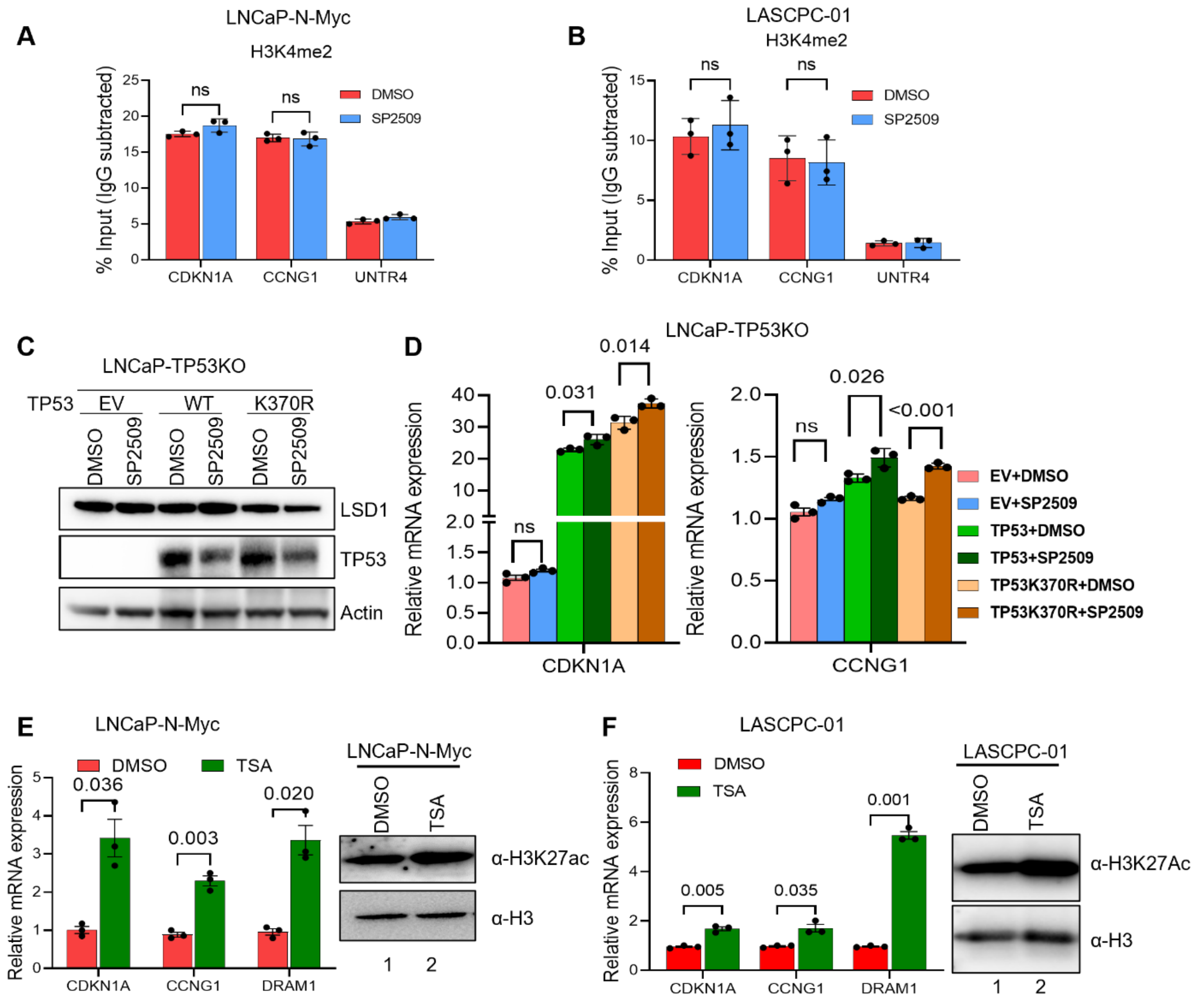

Supplemental Figure 5: HDAC inhibition recapitulates the effects of LSD1 inhibition on TP53 function. *(Related to Figure 5)* (**A-B**) LNCaP-N-Myc (**A**) or LASCPC-01 (**B**) cells were treated with DMSO vehicle or 600 nM SP2509 for 48 hours. ChIP was performed with anti-H3K4me2 antibodies. RT-qPCR was performed to amplify promoter regions of TP53 targets (*CDKN1A, CCNG1*) or a negative control region (UNTR4), n=3. (**C-D**) LNCaP TP53^{KO} cells were reconstituted with either wild-type or demethylation deficient (K370R) TP53 mutant and treated with DMSO vehicle or SP2509 for 48 hours. Expression of TP53 constructs were verified by Western blotting (**C**). Expression of TP53 target genes were analyzed by RT-qPCR (**D**). n=3. (**E-F**) LNCaP-N-Myc (**E**) or LASCPC-01 (**F**) cells were treated with the HDAC inhibitor Trichostatin A (300 nM) and TP53 target gene expression was measured after 24 hours by RT-qPCR. H3K27Ac levels were analyzed by Western blotting (right). Total histone H3 served as a loading control, n=3. For (**A-B** and **D-F**) data are reported as the mean ± SD. For statistical analysis, unpaired two-tailed Student's t-tests were performed, and *P* values are indicated.

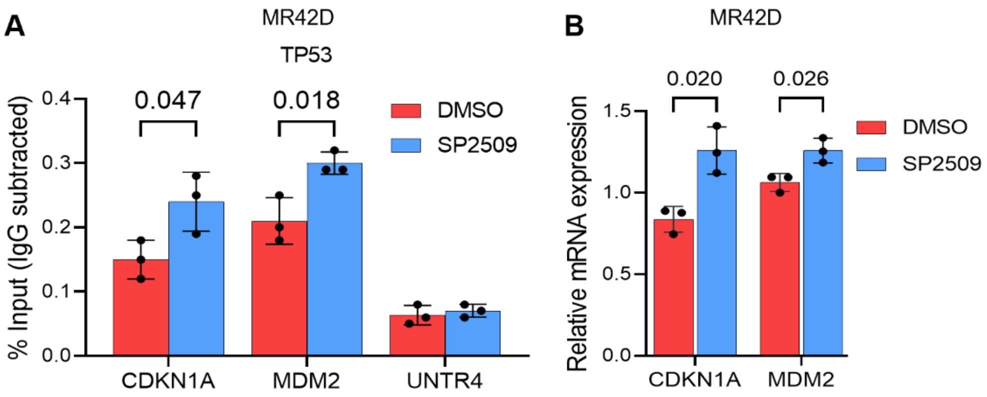

Supplemental Figure 6: LSD1 inhibition increases TP53 occupancy at chromatin. *(Related to Figure 6)* (**A**) MR42D cells were treated with DMSO vehicle or 600 nM SP2509 for 24 hours. ChIP was performed with anti-TP53 antibodies. qPCR was performed to amplify promoter regions of TP53 targets (*CDKN1A, MDM2*) or a negative control region (UNTR4), n=3. (**B**) MR42D cells were treated with DMSO vehicle or 600 nM SP2509 for 24 hours. Expression of TP53 target genes was analyzed by RT-qPCR. All data are reported as the mean ± SD. For statistical analysis, unpaired two-tailed Student's t-tests were performed, and *P* values are indicated.

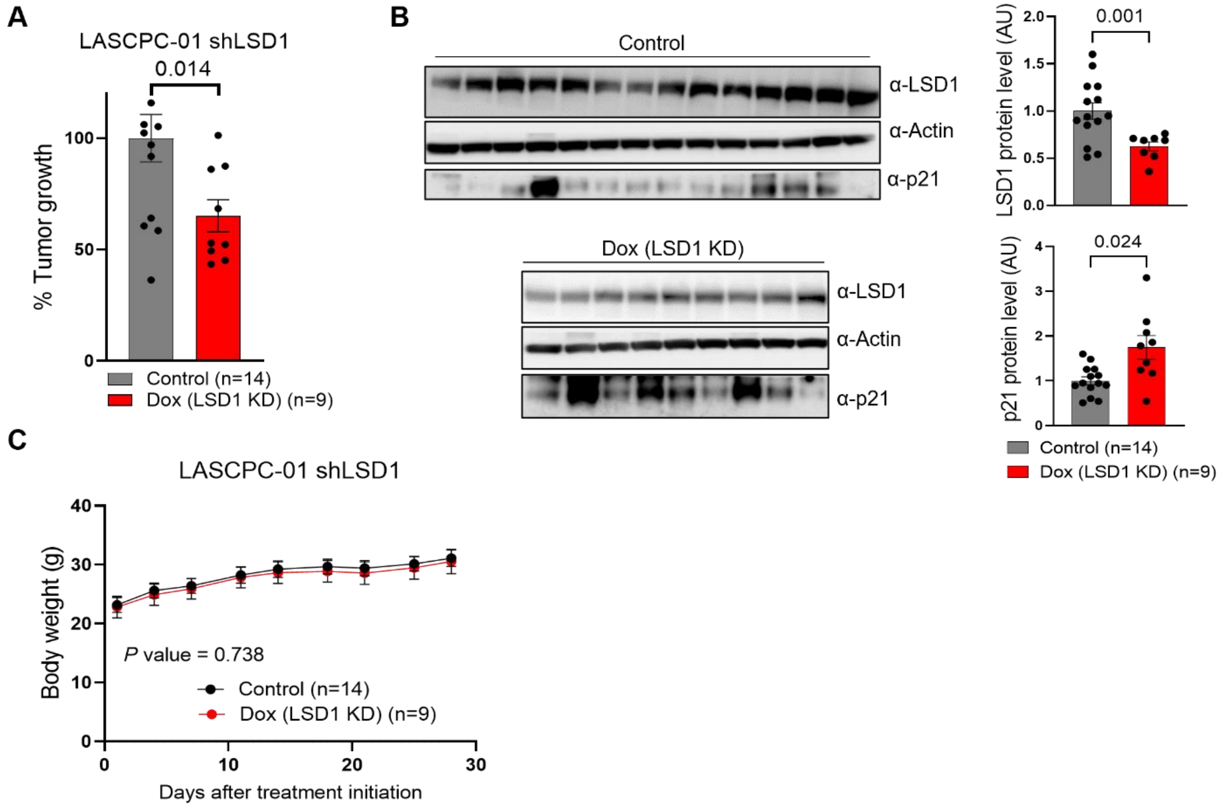

Supplemental Figure 7: LSD1 inhibition suppresses NEPC tumor growth in vivo. *(Related to Figure 7)* (**A**) Tumor volume of LASCPC-01 inducible shLSD1 xenografts in mice treated with control or dox diets was measured at harvest and is presented as a bar graph. The data are reported as the mean ± SEM. For statistical analysis, an unpaired Welch's t-test was performed, and the *P* value is shown. (**B**) Western blot analysis of LASCPC-01 inducible shLSD1 xenografts harvested at endpoint using antibodies specific for LSD1 or p21 (*CDKN1A*). Beta-actin served as a loading control (left). The densitometry analysis of LSD1 and p21 bands are presented as bar plots (right). The data are reported as the mean ± SEM. For statistical analysis, unpaired two-tailed Welch's t-tests were performed, and *P* values are indicated. (**C**) Body weights of mice during the study was measured as a function of time and plotted. The data are reported as the mean ± SEM. For statistical analysis, a mixed-effects model two-way ANOVA was performed, and the *P* value is indicated.

Sample ID	LSD1 score	Subtype
1	271.33	NEPC
2	177.50	NEPC
3	278.33	NEPC
4	190.00	NEPC
5	198.67	NEPC
6	231.67	NEPC
7	157.00	NEPC
8	173.33	NEPC
9	275.67	NEPC
10	226.00	NEPC
11	180.00	NEPC
12	195.00	NEPC
13	156.67	NEPC
14	294.00	NEPC
15	272.67	NEPC
16	290.00	NEPC
17	260.00	NEPC
18	297.33	NEPC
19	298.00	NEPC
20	205.67	Adenocarcinoma
21	197.33	Adenocarcinoma
22	237.50	Adenocarcinoma
23	210.00	Adenocarcinoma
24	230.33	Adenocarcinoma
25	203.33	Adenocarcinoma
26	200.00	Adenocarcinoma
27	204.33	Adenocarcinoma
28	227.50	Adenocarcinoma
29	200.00	Adenocarcinoma
30	60.00	Adenocarcinoma
31	138.33	Adenocarcinoma
32	200.00	Adenocarcinoma
33	103.33	Adenocarcinoma
34	196.67	Adenocarcinoma
35	173.33	Adenocarcinoma
36	197.33	Adenocarcinoma
37	175.00	Adenocarcinoma
38	76.67	Adenocarcinoma
39	156.67	Adenocarcinoma
40	135.00	Adenocarcinoma
41	182.67	Adenocarcinoma
42	192.50	Adenocarcinoma
43	120.00	Adenocarcinoma
44	123.33	Adenocarcinoma

Supplemental Table 1: LSD1 IHC scores of prostate cancer patient tumor samples.

**Gene**	**Taqman assay**	**Species**
CDKN1A	Hs99999142_m1	Human
CCNG1	Hs00171112_m1	Human
DRAM1	Hs01014911_m1	Human
GAPDH	Hs99999905_m1	Human
Cdkn1a	Mm04205640_g1	Mouse
Ccng1	Mm00438084_m1	Mouse
Gapdh	Mm99999915_g1	Mouse

Supplemental Table 2: Taqman assays.

**Gene Symbol**	**Forward Primer Sequence 5'-3'**	**Reverse Primer Sequence 5'-3'**
CDKN1A	CCCGGAAGCATGTGACAATC	CAGCACTGTTAGAATGAGCC
CCNG1	CCTTTTCCACACTAAACTCT	GGACTGGGTCGGCAGACACG
MDM2	TTCAGGGTAAAGGTCACGGG	CCCAATCCCGCCCAGACTAC
UNTR4	CTCCCTCCTGTGCTTCTCAG	AATGAACGTGTCTCCCAGAA

Supplemental Table 3: Primers used for ChIP-qPCR assays.

Supplemental materials and methods

Viability and apoptosis assays

All cell viability measurements were determined using the CellTiter-Glo 2.0 (CTG) assay (Promega cat# G9242) according to the manufacturer's instructions using an Agilent BioTek Synergy H1 Multi-Mode Reader. For the measurement of apoptosis, the RealTime-Glo Apoptosis assay kit (Promega cat# JA1000) was used according to the manufacturer's instructions. Fluorescence measurements were performed using an Agilent BioTek Synergy H1 Multi-Mode Reader.

Immunohistochemistry

Immunohistochemistry (IHC) was performed on 5µm formalin-fixed, paraffin-embedded tissue sections. Metastatic CRPC specimens were collected from patients who died of CRPC and signed written informed consent for a rapid autopsy under the aegis of the Prostate Cancer Donor Program at the University of Washington (IRB protocol # 2341). The Ventana Discovery staining platform with ULTRA Cell Conditioning (ULTRA CC2) solution (Ventana cat# 950-223) was used for antigen retrieval. Immune complexes were developed using the Discovery ChromoMap DAB (diaminobenzidine tetrahydrochloride) Detection Kit (Ventana cat# 760-159). Anti-LSD1 antibody (Cell Signaling Technology, cat# 2139) at 1:250 dilution was used for the IHC assay. The presence and intensity of LSD1 nuclear staining were scored in a blinded fashion. The percentage of tumor cells stained and the staining intensity (strong, 3; moderate, 2; weak, 1; none, 0) were recorded for each case, aggregated as a semi-quantitative product score (range: 0 to 300, Supplemental Table 1), and were plotted using GraphPad Prism version 9.4.1 (GraphPad Software).

Dose-response experiments

For dose-response experiments, indicated cells were treated in biological triplicate for 72 h with a 7-point, 5-fold dilution series from 10 µM of the indicated drugs in DMSO. Cell viability was assessed using the CTG assay. Dose-response was normalized to the vehicle-treated growth rate and fitted with a logistic curve as previously described (1).

LSD1 inhibition assay

The LSD1 inhibition assay was performed using the LSD1 Inhibitor Screening Assay Kit (Cayman Chemical, cat# 700120) using the manufacturer's protocol. LSD1 activity was measured in the presence of DMSO vehicle or the LSD1 inhibitors GSK2870552, GSK-LSD1, SP2509, and SP2577.

Cell cycle analysis

For cell cycle analysis, cells were harvested in hypotonic propidium iodide buffer (0.1% Sodium citrate, 0.1% Triton-X100, 100 µg/mL RNase A, and 50 µg/mL propidium iodide) and incubated at room temperature for 5 minutes. The cells were analyzed using a BD LSRFortessa Cell Analyzer (BD Biosciences), and data were analyzed using FlowJo version 10.8.1 (BD Biosciences) to obtain the percentage of cells in each phase of the cell cycle. The data was used to plot graphs using GraphPad Prism version 9.4.1.

Plasmid Transfection:

For overexpression experiments, plasmids were transfected using Lipofectamine 3000 reagent (Thermo Fisher cat# L3000008) per manufacturer's recommendations. Cells were harvested 48 hours post-transfection and processed for downstream analyses. LSD1 expression constructs have been described previously (2). *TP53* constructs were a kind gift from Basant Kumar Thakur, University Hospital Essen, Essen, Germany (3).

Transient knockdown experiments

Transient knockdowns were performed using siRNA oligonucleotides or shRNA constructs described previously (2). The siRNA oligonucleotides were transfected with DharmaFECT 3 (GE Dharmacon) transfection reagent for 96 h. Cells used for RNA and protein harvest were seeded and transfected in 6-well plates. Cells used in viability assays were seeded and transfected in 96-well plates. Cell viability was measured at time 0 and endpoint using the CTG assay; these values were used to calculate the relative growth rate as described previously (4).

RNA preparation and RT-qPCR

After the indicated treatments, RNA was extracted from cells using the RNeasy Plus Mini Kit (Qiagen cat# 74034) according to the manufacturer's protocol. After RNA extraction, 1 µg RNA was reverse-transcribed into cDNA using the High-Capacity cDNA Reverse Transcription kit (Life Technologies cat# 4368814) with random hexamer primers. RT-qPCR was performed using a Quantstudio 3 or Quantstudio 12 thermocycler (Life Technologies) with the following program: 50°C for 2 min, 95°C for 10 min, and 40 cycles of 95°C for 15 sec dissociation, 60°C for 1 min annealing/extension/read. 10 µL singleplex RT-qPCR reactions contained 1X TaqMan universal PCR master mix (Thermo Fisher Scientific cat# 4304437), 1X Primer and Taqman hydrolysis probe specific to the target tested (Supplemental Table 2), and 10 ng RNA-equivalent cDNA templates. *GAPDH* was used as endogenous control. Data were analyzed with Design and Analysis Software version 1.5.2 (Life Technologies).

Western blotting

Western blotting experiments were performed by running protein lysates on SDS-PAGE gels (Thermo Fisher Scientific cat# NP0335BOX) and transferring them onto PVDF membranes as described previously (4). Blots were probed with indicated antibodies and imaged using a Chemidoc MP imaging system (Bio-Rad). Densitometry analysis was performed using NIH Image J (5).

Co-immunoprecipitation

For co-immunoprecipitation experiments, cell lysates were prepared in RIPA buffer (Thermo Fisher Scientific cat # 89900) with protease inhibitors (Thermo Fisher Scientific cat# A32965). The cell lysates were centrifuged at 16,000 × g for 20 min at 4°C. The supernatants were collected and precleared with protein G-conjugated Dynabeads (Thermo Fisher Scientific cat# 10004D) for 1 h at 4°C. SP2509 was added to the precleared supernatant at a final concentration of 600 nM and then incubated with indicated antibodies on a rotary platform at 4°C for overnight. Then protein G-conjugated Dynabeads were added and further incubated for 2 h on a rotary platform at 4°C. The unbound supernatant was aspirated from the beads after sedimenting the beads on a magnetic stand, and the beads were washed five times with ice-cold IP buffer (Thermo Fisher Scientific cat# 87787). The immunoprecipitated protein complexes were eluted by adding of 35 µL of SDS-PAGE loading buffer. Eluted samples and input fractions were analyzed by immunoblots.

Chromatin immunoprecipitation (ChIP)

ChIP experiments were performed as described previously (6) using the specified antibodies or rabbit IgG (Millipore cat# 12-370). Briefly, 10 million formaldehyde cross-linked cells were lysed and sonicated with the Bioruptor Pico (Diagenode). 4 µg antibody was used to immunoprecipitate chromatin from 2 million cells per ChIP overnight at 4°C. The DNA–protein–antibody complexes were collected with 30 µL resuspended Protein A/G magnetic beads (ThermoFisher Scientific cat#26162). After washing, DNA was extracted by 10% Chelex-100 resin (Bio-Rad cat# 1421253) and digested with Proteinase K (Invitrogen cat# AM2546). qPCR was performed as described above with SYBRGreen PCR master mix (Thermo Fisher Scientific cat# 4312704) using a Quantstudio 3 or Quantstudio 12K Flex thermocycler (Thermo Fisher Scientific). Primer sequences are provided in Supplemental Table 3.

In vivo anti-tumor activity of LSD1 suppression

All the studies were performed in 6-8 week-old male athymic homozygous nude-Foxn1nu mice (Jackson Laboratories cat# 002019). All cells for implantation were prepared in 1:1 growth media/Matrigel (Corning cat# 356234) mixture. One million cells (LASCPC-01 or MR42D) were

implanted subcutaneously (s.c.) in mice. Once tumors reached ~100 mm^3, mice were randomized into vehicle (10% DMSO, 30% Solutol, 60% water) or treatment (150 mg/kg SP2577 in vehicle) groups and dosed by oral gavage twice a day (PO BID) for two (MR42D) or three (LASCPC-01) weeks. Animals were sacrificed if the tumors reached the humane endpoint according to the approved protocol before treatment completion. For the MR42D experiment, mice were castrated and additionally dosed with 100 mg/kg enzalutamide by oral gavage from the day before cell implantation (7). For the inducible LSD1 knockdown experiment, 1 million LASCPC-01 cells stably expressing dox-inducible LSD1 shRNA were implanted s.c. in mice. Immediately after injection, the mice were randomized to the control arm (normal diet, Bioserv cat# S4207) or dox arm (diet containing doxycycline, Bioserv cat# S3888). All animals were sacrificed on day 28. Body weight and tumor measurements for all experiments were recorded twice weekly.

RNA-seq

Total RNA was extracted with RNeasy Plus Mini Kit as above. Library preparation and sequencing methods were performed as described previously (4). The libraries were barcoded, pooled, and sequenced using paired-end 151 bp (LNCaP-N-Myc cells treated with vehicle or SP2509) or paired-end 100 bp (LASCPC-01 and MR42D cells treated with vehicle or SP2509) sequencing. Reads were mapped to GRCh38 using STAR version 2.5.2a (8), and gene quantifications were calculated using Stringtie version 2.1.1 (9-11) to quantify RefGene annotations. Gene read counts calculated using featureCounts (12) version 1.6.1 (subread) were used to evaluate differential expression using DESeq2 version 1.30.1 (13). Gene Set Enrichment Analysis (GSEA) was performed using command line GSEA version 4.1.0 (14,15) using ranked shrunkLFC (shrunk Log Fold Change) of genes. Over Representation Analysis (ORA) was performed using WebGestaltR version 0.4.4 (16-19), using protein-coding genes with Padj ≤ 0.05 and absolute shrunkLFC ≥ log2(1.5).

Master regulator and pathway analysis

RNA-seq data from cell lines treated with vehicle or SP2509 were used to perform pathway analysis and to evaluate differential transcription factor activities. Differential gene expression analysis between experimental groups was first performed using DESeq2 (13). Gene expression differences were considered significant with Padj < 0.05. The Wald test statistic results from DESeq2 were used to perform pathway and master regulator analyses. The collections of gene sets were downloaded from version 7.4 of the Molecular Signatures Database (MSigDB; https://www.gsea-msigdb.org/gsea/msigdb/). Pathway enrichment analysis was computed by the pre-ranked version of the CAMERA method, the cameraPR function, implemented in the limma R package (version 3.48.3) (20). Master regulator analysis was performed by msVIPER algorithms provided in the VIPER R package (version 1.26.0) (21). The transcription factor

regulons (the regulatory network) used in this study were curated from four databases as previously described (22). ARG10 (23) and NEPC Up (24) signatures were derived from previously reported datasets.

TP53 signature analysis

The TP53 signature was described previously by Chipidza et al. (25). The TP53-WT Centroid genes of the TP53 signature were used to build a pseudo-regulon of TP53. Forty-one up-regulated and 145 down-regulated genes served as positive and negative target genes of the pseudo-regulon of TP53, respectively. To measure the TP53 regulon activity in each sample, we used the VIPER algorithm (21) implemented in the VIPER R package (version 1.26.0). A log1*P* transformed TPM gene expression matrix and a regulatory network were used as inputs for VIPER analysis. The viper function was employed to calculate the regulon activities of the TP53 signature and other transcription factors on different datasets. The regulatory network used in VIPER analysis was the same as described above.

Supplemental References:

1. Hafner M, Heiser LM, Williams EH, Niepel M, Wang NJ, Korkola JE*, et al.* Quantification of sensitivity and resistance of breast cancer cell lines to anti-cancer drugs using GR metrics. Sci Data **2017**;4:1-9
2. Sehrawat A, Gao L, Wang Y, Bankhead A, 3rd, McWeeney SK, King CJ*, et al.* LSD1 activates a lethal prostate cancer gene network independently of its demethylase function. Proc Natl Acad Sci U S A **2018**;115:E4179-E88
3. Bruer M, Reinhardt D, Welte K, Thakur BK. Insights into the limitations of transient expression systems for the functional study of p53 acetylation site and oncogenic mutants. Biochem Biophys Res Commun **2020**;524:990-5
4. Kim DH, Sun D, Storck WK, Welker Leng K, Jenkins C, Coleman DJ*, et al.* BET Bromodomain Inhibition Blocks an AR-Repressed, E2F1-Activated Treatment-Emergent Neuroendocrine Prostate Cancer Lineage Plasticity Program. Clin Cancer Res **2021**;27:4923-36
5. Schneider CA, Rasband WS, Eliceiri KW. NIH Image to ImageJ: 25 years of image analysis. Nat Methods **2012**;9:671-5
6. Nelson JD, Denisenko O, Bomsztyk K. Protocol for the fast chromatin immunoprecipitation (ChIP) method. Nat Protoc **2006**;1:179-85
7. Clegg NJ, Wongvipat J, Joseph JD, Tran C, Ouk S, Dilhas A*, et al.* ARN-509: a novel antiandrogen for prostate cancer treatment. Cancer Res **2012**;72:1494-503
8. Dobin A, Davis CA, Schlesinger F, Drenkow J, Zaleski C, Jha S*, et al.* STAR: ultrafast universal RNA-seq aligner. Bioinformatics **2013**;29:15-21
9. Kovaka S, Zimin AV, Pertea GM, Razaghi R, Salzberg SL, Pertea M. Transcriptome assembly from long-read RNA-seq alignments with StringTie2. Genome Biol **2019**;20:278
10. Pertea M, Kim D, Pertea GM, Leek JT, Salzberg SL. Transcript-level expression analysis of RNA-seq experiments with HISAT, StringTie and Ballgown. Nat Protoc **2016**;11:1650-67
11. Pertea M, Pertea GM, Antonescu CM, Chang TC, Mendell JT, Salzberg SL. StringTie enables improved reconstruction of a transcriptome from RNA-seq reads. Nat Biotechnol **2015**;33:290-5
12. Liao Y, Smyth GK, Shi W. featureCounts: an efficient general purpose program for assigning sequence reads to genomic features. Bioinformatics **2014**;30:923-30
13. Love MI, Huber W, Anders S. Moderated estimation of fold change and dispersion for RNA-seq data with DESeq2. Genome Biol **2014**;15:550
14. Subramanian A, Tamayo P, Mootha VK, Mukherjee S, Ebert BL, Gillette MA*, et al.* Gene set enrichment analysis: a knowledge-based approach for interpreting genome-wide expression profiles. Proc Natl Acad Sci U S A **2005**;102:15545-50
15. Mootha VK, Lindgren CM, Eriksson KF, Subramanian A, Sihag S, Lehar J*, et al.* PGC-1alpha-responsive genes involved in oxidative phosphorylation are coordinately downregulated in human diabetes. Nat Genet **2003**;34:267-73
16. Liao Y, Wang J, Jaehnig EJ, Shi Z, Zhang B. WebGestalt 2019: gene set analysis toolkit with revamped UIs and APIs. Nucleic Acids Res **2019**;47:W199-W205
17. Wang J, Vasaikar S, Shi Z, Greer M, Zhang B. WebGestalt 2017: a more comprehensive, powerful, flexible and interactive gene set enrichment analysis toolkit. Nucleic Acids Res **2017**;45:W130-W7
18. Wang J, Duncan D, Shi Z, Zhang B. WEB-based GEne SeT AnaLysis Toolkit (WebGestalt): update 2013. Nucleic Acids Res **2013**;41:W77-83
19. Zhang B, Kirov S, Snoddy J. WebGestalt: an integrated system for exploring gene sets in various biological contexts. Nucleic Acids Res **2005**;33:W741-8
20. Ritchie ME, Phipson B, Wu D, Hu Y, Law CW, Shi W*, et al.* limma powers differential expression analyses for RNA-sequencing and microarray studies. Nucleic Acids Res **2015**;43:e47

21. Alvarez MJ, Shen Y, Giorgi FM, Lachmann A, Ding BB, Ye BH*, et al.* Functional characterization of somatic mutations in cancer using network-based inference of protein activity. Nat Genet **2016**;48:838-47
22. Robertson AG, Shih J, Yau C, Gibb EA, Oba J, Mungall KL*, et al.* Integrative Analysis Identifies Four Molecular and Clinical Subsets in Uveal Melanoma. Cancer Cell **2017**;32:204-20 e15
23. Bluemn EG, Coleman IM, Lucas JM, Coleman RT, Hernandez-Lopez S, Tharakan R*, et al.* Androgen Receptor Pathway-Independent Prostate Cancer Is Sustained through FGF Signaling. Cancer Cell **2017**;32:474-89 e6
24. Beltran H, Prandi D, Mosquera JM, Benelli M, Puca L, Cyrta J*, et al.* Divergent clonal evolution of castration-resistant neuroendocrine prostate cancer. Nat Med **2016**;22:298-305
25. Chipidza FE, Alshalalfa M, Mahal BA, Karnes RJ, Liu Y, Davicioni E*, et al.* Development and Validation of a Novel TP53 Mutation Signature That Predicts Risk of Metastasis in Primary Prostate Cancer. Clin Genitourin Cancer **2021**;19:246-54 e5
26. Aggarwal R, Huang J, Alumkal JJ, Zhang L, Feng FY, Thomas GV*, et al.* Clinical and Genomic Characterization of Treatment-Emergent Small-Cell Neuroendocrine Prostate Cancer: A Multi-institutional Prospective Study. J Clin Oncol **2018**;36:2492-503
27. Aggarwal RR, Quigley DA, Huang J, Zhang L, Beer TM, Rettig MB*, et al.* Whole-Genome and Transcriptional Analysis of Treatment-Emergent Small-Cell Neuroendocrine Prostate Cancer Demonstrates Intraclass Heterogeneity. Mol Cancer Res **2019**;17:1235-40
28. Abida W, Cyrta J, Heller G, Prandi D, Armenia J, Coleman I*, et al.* Genomic correlates of clinical outcome in advanced prostate cancer. Proc Natl Acad Sci U S A **2019**;116:11428-36
29. Colaprico A, Silva TC, Olsen C, Garofano L, Cava C, Garolini D*, et al.* TCGAbiolinks: an R/Bioconductor package for integrative analysis of TCGA data. Nucleic Acids Res **2016**;44:e71
